# The use and misuse of the SCOFF screening measure over two decades: a systematic literature review

**DOI:** 10.1007/s40519-024-01656-6

**Published:** 2024-04-23

**Authors:** Amy Coop, Amelia Clark, John Morgan, Fiona Reid, J. Hubert Lacey

**Affiliations:** 1 Schoen Clinic Newbridge, Birmingham, UK; 2 Schoen Clinic Chelsea, London, UK; 3 St George’s, University of London, London, UK; 4 King’s College, London, UK

**Keywords:** SCOFF, Eating disorders, Anorexia nervosa, Bulimia nervosa, Questionnaires, Screening

## Abstract

**Purpose:**

The SCOFF questionnaire was designed as a simple, memorable screening tool to raise suspicion that a person might have an eating disorder. It is over 20 years since the creation of the SCOFF, during which time it has been widely used. Considering this, we wish to review the use of the SCOFF in peer-reviewed scientific journals, and to assess whether it is being used appropriately in the manner in which it was originally devised and tested.

**Methods:**

The Preferred Reporting Items for a Systematic Review and Meta-analysis (PRISMA) guidelines were followed, and all search strategies and methods were determined before the onset of the study. PubMed and Wiley Online Library were searched using the terms *SCOFF* and *eating*. Two reviewers were involved in the reviewing process. Criteria for appropriate use of the SCOFF were formalised with the tool’s original authors.

**Results:**

180 articles were included in the final review. 48 articles had used the SCOFF appropriately, 117 articles inappropriately and 15 articles had been mixed in the appropriateness of their use.

**Conclusion:**

This systematic review highlights the inappropriate use of the SCOFF in diverse languages and settings. When used correctly the SCOFF has made a significant contribution to the understanding of eating disorders and its simplicity has been applauded and led to widespread use. However in over two-thirds of studies, the use of the SCOFF was inappropriate and the paper highlights how and in what way it was misused, Guidelines for the appropriate use of the SCOFF are stated. Future validation and avenues of research are suggested.

**Level of evidence:**

Level I.

**Supplementary Information:**

The online version contains supplementary material available at 10.1007/s40519-024-01656-6.

## Introduction

Early detection and treatment of eating disorders (EDs) can improve prognosis and likelihood of recovery [1], however their presence can often be overlooked or misdiagnosed initially by clinicians [2]. With an estimated 55.5 million people affected [3] and rates appearing to be on the rise [4, 5], the use of tools to assist in the timely identification and access to support for ED sufferers is important.

The SCOFF questionnaire, first published in 1999, was designed as a simple, memorable screening tool to raise suspicion that an ED might exist [6]. It was developed in response to a lack of robust short screening measures, particularly for use in primary care [7]. The questionnaire was intended to suggest a likely case, rather than to diagnose an ED, and to be followed up by a thorough assessment with a trained clinician prior to diagnosis [6]. It was validated against the DSM-IV diagnostic criteria for anorexia nervosa (AN) and bulimia nervosa (BN) [6]. The measure has been used widely since its conception in both clinical and research settings and has gained traction in popular culture with features in publications such as Teen Vogue [8] and GQ magazine [9]. However, we have become aware of some examples of the inappropriate use of the SCOFF as a diagnostic rather than screening tool; for example, it was recently employed as part of the NHS Health Survey for England to estimate the prevalence of EDs in the country [10].

It is over 20 years since the SCOFF was devised and tested and we, the original developers, conscious of its widespread use in many countries and languages, feel it timely to review its use in peer-reviewed scientific journals. This paper reviews a large sample of the available literature and assesses the appropriateness of the SCOFF’s use in each publication. The review will not be addressing the SCOFF’s misuse amongst non-research settings (e.g. public health campaigns), however it will aim to clarify the guidelines of using the SCOFF, thereby benchmarking the future use of the SCOFF. To the authors’ knowledge, there have not been similar reviews assessing the appropriate use of the SCOFF questionnaire.

## Method

### Search strategy and inclusion criteria

The Preferred Reporting Items for a Systematic Review and Meta-analysis (PRISMA) guidelines [11] were followed in preparing this systematic review and all search strategies and methods were determined before the onset of the study. The review was not registered. The intention to conduct the review was announced at a national and an international conference and it was agreed by the original authors and the editor of a potential journal. The intention was stated on the UK ED specialist website to avoid unnecessary duplication.

**Fig. 1:**
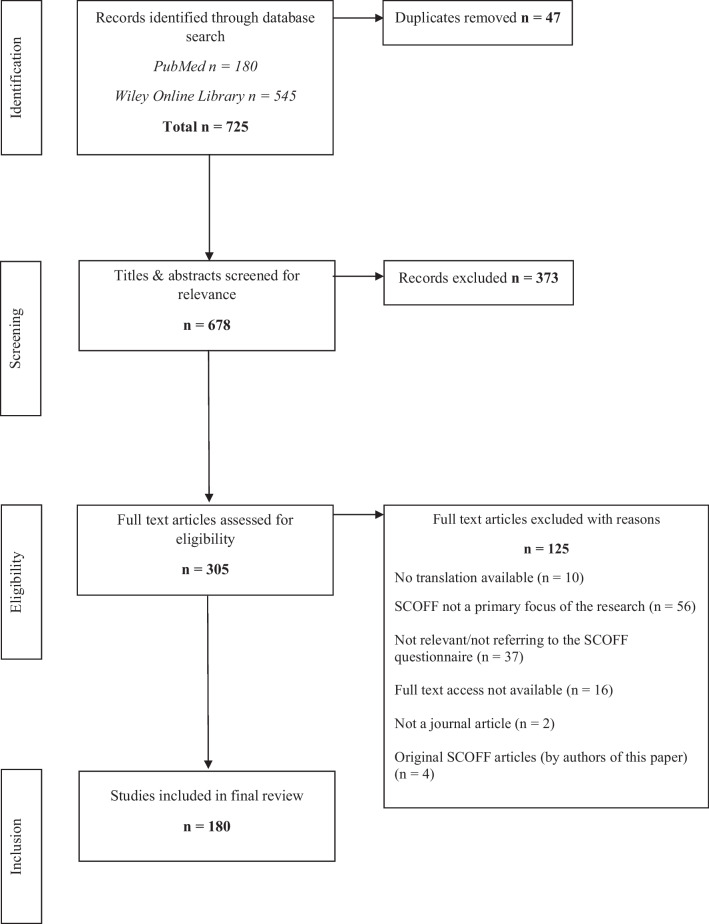
PRISMA flowchart

For the literature search, PubMed and Wiley Online Library were employed. The publication timeframe was limited to 1999 (the year the questionnaire was developed) through to the date of the search. The search terms *SCOFF* and *eating* were searched for in the title and/or abstract. It was felt these search terms captured the breadth of the literature whilst excluding many publications which used the term ‘scoff’ in the colloquial sense. The results from the search are given in Fig. 1.

Two reviewers (ACo and ACl) were involved in the search process and the results were considered in two steps. Initially, the titles and abstracts of each article were reviewed, by either ACo or ACl, using the following inclusion criteria: (1) a journal article published in an academic journal; (2) the article was written in English and/or a translation was available; (3) the article was related to eating and/or mental health. The full text of those articles included were then examined. During the full review articles were required to meet the initial inclusion criteria and, in addition, demonstrate use of the SCOFF questionnaire as a primary focus within their research (i.e. not simply as a reference). To avoid conflicts of interest, articles authored by the original SCOFF authors in relation to the development of the tool were excluded. Full text articles were reviewed twice—once by each reviewer to ensure the review was robust and objective. Finally, articles included in the final review were assessed to be either appropriate or inappropriate in their use of the SCOFF. It was felt that a subsection of articles were less clear cut in the appropriateness of their use of the measure. These articles tended to have used the SCOFF appropriately, however could have ideally used more caution in their interpretation of the results or been more clear and consistent in their description of the measure and/or results. As such, these were classified as ‘mixed’ use. The classification of each article was cross-checked by each reviewer independently. Any uncertainties at any stage of the review process were resolved by the SCOFF’s original authors.

### Data extraction and quality assessment

The reviewers (ACo and ACl) used a data collection tracker to extract data on the title, database extracted from, author(s), year of publication, research study design, aim of the study, the use of the SCOFF questionnaire, and the appropriateness of its use. Criteria for appropriate use—which were developed with the tool’s original authors—are shown in Table 1.

**Table 1:** Criteria for appropriate use of the SCOFF

All articles	Must be utilised as a screening tool, not diagnostic
	Must interpret results as a ‘screen’ (e.g. ‘screened positive’)
	Must include the term ‘eating disorder’ but with a level of caution (e.g. ‘possible/likely eating disorder’ or ‘at increased risk of having an ED’)
Translated/modified versions	Needs to have been back translated or used another appropriate translation method
	Needs to be some evidence of validation of the modified version
Amending scoring	The cut-off point of 2 can be altered in different populations if a clinical rationale is presented and there is evidence of validation of the amended measure

The quality of the research articles included in this systematic review was not analysed, beyond looking at the appropriate use of the SCOFF, as it was important for articles of all quality to be included to assess the full breadth of research.

## Results

A full overview of the articles reviewed and details of the findings can be found in the supplementary table (Supplementary Information). One hundred and eighty articles met the criteria for inclusion in the review (Fig. 1). Forty-eight articles had used the SCOFF tool appropriately [12–59], 117 articles had used the SCOFF inappropriately [60–176] and 15 articles had been mixed in their use [177–191]. The review found 16 translations of the SCOFF, including Italian, Brazilian-Portuguese, Arabic and Chinese (more information can be found in the Supplementary Information). Table 2 gives a summary of the usage errors in the ‘not appropriate’ and ‘mixed’ categories. A subsection of articles fell under multiple usage errors (see Supplementary Information for further details).

**Table 2:** Description of usage errors of articles classed as not appropriate or mixed use of the SCOFF

	Usage error	Number of articles
A	Insufficient caution used when interpreting the results, resulting in a more diagnostic interpretation than screening	46
B	Used to measure ‘disordered eating’ (abnormal eating behaviour that does not meet the criteria for a clinically diagnosable ED [192]) or similar	34
C	Used to assess ED symptoms, either as a continuous 5-point scale or via analysis of separate items	26
D	Incorrect interpretation of how SCOFF relates to the risk of having an ED (including interpreting as future risk; or a negative SCOFF meaning no risk; or an imprecise/ambiguous description of risk)	20
E	Inconsistent interpretation of the SCOFF as a screening tool throughout the article	17
F	Amended scoring/cut-off thresholds for the SCOFF but without clinical rationale given	9
G	No clear description of the SCOFF as a screening tool	4
H	No validation of a translated measure	3
I	Used to assess body dysmorphic disorder, without validation	1

107 papers were attributed to one error, 22 papers to 2 errors, 3 papers to 3 errors

## Discussion

Early detection of EDs is an important factor for improved prognosis [1]. The SCOFF questionnaire was developed primarily for use, as a screening tool in primary care, to facilitate early detection of EDs [7]. This review examined the widespread use of the SCOFF questionnaire in the academic field. Its use has assisted in expanding knowledge and the understanding of eating disorders. However, it is clear from this review that the original function of the tool, that is, to raise suspicion that an eating disorder may be present, has in many cases been lost. Researchers, clinicians and peer-reviewed journals need to be more rigorous in only accepting data when the SCOFF is used as described in the original studies which reported its efficacy and reliability. This should not preclude, however, encouraging some exciting avenues of the SCOFF’s use which require further research, such as the validation of SCOFF for DSM-V criteria for EDs, and the use of the SCOFF to assess risk of future EDs. To assist, we have provided guidelines for appropriate use of the SCOFF in Table 1.

This review shows that the use of the SCOFF has grown globally and has been translated into many different languages. Overall, the translation of the SCOFF was executed well with the use of back-translation methods, or similar, and for most translations the modified version was further validated. The vast translation and global use of the SCOFF reflects its memorability and simplicity.

The authors found that the level of caution used when reporting and interpreting the SCOFF results was insufficient and/or inconsistent in a large number of articles. Researchers must remain mindful as to the screening nature of the SCOFF and report their results in line with this. It is paramount that the reader is clear the SCOFF merely raises the suspicion that an ED may exist, as opposed to confirming its presence as a diagnostic fact.

The SCOFF was at times employed to assess a persons’ future risk of developing an ED, which is distinctly different to assessing the likelihood that an ED may exist currently. The items on the SCOFF tool address the core features of AN and BN. To measure a person’s future risk of developing an ED, greater focus on risk factors is needed [192]. Additionally, the SCOFF has not been validated against future risk and as such this use should be avoided until further validation has been completed. Some articles mistakenly interpreted a negative SCOFF result as indicating ‘no risk of ED’, while others were imprecise or unclear in their use of the terminology around risk. Other articles presented the ‘risk of EDs’ for their whole sample group—this is effectively prevalence, and reflects SCOFF being used inappropriately in a diagnostic tool.

There is perhaps a more philosophical question around the meaningfulness of studies looking for correlates or predictors of being screened positive on SCOFF (i.e. using an endpoint of ‘positive/negative screen’ or ‘at higher risk of having an ED/not higher risk’). Even when sufficient caveats were provided on the exact meaning of being SCOFF positive, in most cases it seemed that this was being presented as a proxy for having an eating disorder. The use of SCOFF (or indeed any screening tool) to define an endpoint in its own right goes beyond its original purpose and requires careful consideration in studies planning such use.

The SCOFF was also widely used to assess disordered eating (DE). DE is generally considered to refer to abnormal eating behaviour that does not meet the criteria for a clinically diagnosable ED [193]. Although the SCOFF is designed to be supplemented by further clinical assessment, it was developed in line with the core features of AN and BN and was validated against DSM-IV diagnostic guidelines [6]. As such, the SCOFF has not been validated to assess eating behaviour that does not meet the diagnostic threshold. From the articles included in this review, there does not appear to have been a robust validation of the SCOFF for assessment of DE, however this would be an exciting avenue of research and would further strengthen the tool’s use for early detection within primary care.

The use of the SCOFF to assess ED symptoms was also present in a subsection of the articles reviewed. A number of these articles analysed the individual items of the SCOFF separately to explore specific ED symptoms. Others analysed the SCOFF as a continuous measure (i.e. a higher score indicated more ED symptoms). Again, authors must remain mindful as to the screening nature of the SCOFF. Consisting of just five items, the SCOFF should be taken as a whole, categorical measure to maintain the specificity and sensitivity of the tool. In addition, the original purpose of the tool must be maintained—that is, to raise suspicion that an ED may exist as opposed to assessing the level or presence of symptomatology.

When the SCOFF is used outside of the parameters of its validity and reliability there is a far higher probability of the outcomes being misinterpreted and overstated—as was the case with the media’s coverage of the NHS Health Survey for England [194]. Thus, it is important to ensure the measure is being used appropriately. Nonetheless, it is promising to see an easily accessible screening tool, such as the SCOFF, used so widely within academia and an ever-growing body of research into EDs. There are some exciting avenues of research to explore expanding the scope of the SCOFF. However, in the absence of further validation, the authors of this review, and the original authors of the SCOFF, propose the criteria for appropriate use of the questionnaire detailed in the methodology of this review (Table 1) be used at present to preserve its function as a screening tool.

### Strengths and limitations

The primary strength of this review is the number of articles included due to the wide scope of the search strategy. In addition, the inclusion of a secondary reviewer ensured a robust methodology and limited the subjectivity of the results. Due to the vast amount of research employing the SCOFF and the limited manpower of this review, the decision was made to limit the search to just two databases and not to conduct further stages of the search process such as manually searching reference lists. In addition, due to access rights, the reviewers were limited in the databases they were able to search, hence why PubMed and Wiley Online Library were selected. The authors acknowledge that, as such, a subsection of relevant articles may have been missed, however it is felt that the objective of the review was achieved nevertheless, and the review remained systematic in nature due to the methods employed.

### Conclusion

This paper reviewed and assessed the use of the SCOFF questionnaire in a large sample of academic papers. In two-thirds of cases, researchers used the SCOFF inappropriately and this paper highlights how and in what way it was misused, However, when used correctly (using the guidelines given in Table 1), the SCOFF remains a relevant, valid and reliable tool for the screening of possible ED cases. Its use within the research field and beyond is indicative of the ever-growing exploration into and understanding of these disorders. There are exciting avenues of future research identified in this review which the authors encourage, including validation of the SCOFF against updated DSM-5 criteria, its ability to assess future risk, and its efficacy in screening for DE.

## What is already known on this subject?

The use of the SCOFF is commonplace within academic literature. The original article detailing the development of the SCOFF outlined its intended use.

## What this study adds?

This review shows that in many peer-reviewed papers, the SCOFF is used inappropriately and the paper details how and in what way it is misused. The paper re-establishes guidelines for the SCOFF’s appropriate use.

### Supplementary Information

Below is the link to the electronic supplementary material.

Supplementary file 1 (PDF 529 KB)

## Data Availability

Detailed data availability is in the supplementary table available on the online version.
