# Supplementary material for: The use and misuse of the SCOFF screening measure over two decades: a systematic literature review
Source: Eat Weight Disord. 2024 Apr 23;29(1):29. doi: 10.1007/s40519-024-01656-6 (PMC11039549; doi:10.1007/s40519-024-01656-6)
Supplement: Supplementary file 1 — (PDF 529 KB) [file 40519_2024_1656_MOESM1_ESM.pdf]

The use and misuse of the SCOFF screening measure over two decades: A systematic literature review

Amy Coop, Schoen Clinic Newbridge, Birmingham, UK

Amelia Clark, Schoen Clinic Chelsea, London, UK

Prof. John Morgan, St George's, University of London, London, UK

Fiona Reid, King's College London, London, UK

Prof. J Hubert Lacey, St George's, University of London, London, UK ORCID: 0000-0001-7302-6496

Online Resource 1: Full analysis of included studies

Authors	Type of study	Aim of study	Use of the SCOFF	Version used	Final categorization	Error Code (see Table 2)	Rationale for categorization
Coffino & Hormes (2018)[13]	RCT	To examine the feasibility and initial efficacy of a novel default option intervention targeting nutritional quality of online grocery purchases within the financial constraints of food insecurity.	One of the exclusion criteria of the RCT was "no current ED diagnosis, as assessed via the SCOFF screening measure (with a score ≥2 considered indicative of the likely presence of an ED)"	Original	Not appropriate	B	Using the SCOFF to assess the exclusion criteria of 'no current ED diagnosis'

Pannocchia et al. (2011)[14]	Validation	To explore the psychometric properties of an Italian translation of the SCOFF	The SCOFF was translated into Italian using the back-translation method. It was then initially administered to 20 subjects to identify any unclear questions. All questions turned out to be clear and understandable. The Italian translation of the SCOFF was administered, together with a part of the Italian version of the EDs Inventory-3, to a sample of psychiatric patients diagnosed with ED (according to the DSM-IV), a sample of female psychiatric controls with no ED symptoms, and a sample of female normal controls without EDs from the general population.<br>Construct validity, concurrent validity and reliability (inter-item total correlations and Cronbach's alpha) were assessed.	Italian	Appropriate		Robust translation and validation method
Mari et al. (2018)[15]	Prospective study	To assess the correlation between compliance with a lowFODMAP diet and the risk of ED behaviours among patients with IBS	Used to assess participants risk for ED behaviour. A score of 2 or greater indicated an increased risk of ED behaviour.	Original	Not appropriate	A	The SCOFF has not been validated to assess a person's *risk* of developing ED behaviours
Kenny et al. (2018)[16]	Crosssectional study	To compare insomnia symptoms in individuals with BED and those with no history of an ED (NED)	Individuals were ineligible to participate in the NED group if they reported a current or previous ED diagnosis or exceeded a cut-off of 3 on the SCOFF.<br>Described the SCOFF a 5-item yes or no screening questionnaire for the identification of individuals at risk of an ED	Original	Not appropriate	B F	Using the SCOFF to assess the exclusion criteria of 'no current ED diagnosis'<br>No clinical rationale given for amended scoring criteria

Leung, Joyce & Russell (2013)[17]	Open trial	To evaluate the self-help behaviour of individuals with EDs in an Internet-based selfhelp programme developed in the AsiaPacific region and to determine their compliance with the programme	Used to assess participants eating disorder psychopathology. A score of ≥2 on the SCOFF indicated high risk for an ED. If participants did not score significantly on EDE-Q but scored ≥2 on the SCOFF they were diagnosed with a clinically significant ED.	Original	Not appropriate	A B	The SCOFF has not been validated to assess a person's *risk* of developing an ED. The SCOFF should not be used to diagnose clinically significant EDs.
Melchior et al. (2020)[18]	Crosssectional study	To compare the prevalence of anxiety and depression states and EDs between patients with irritable bowel syndrome (IBS) and healthy volunteers without IBS.	Used to screen participants for ED in those with IBS and those without. Used French version (validated and back translated).	French	Not appropriate	B	Results are not cautious enough in relation to the screening nature of the tool. (e.g., "IBS patients with ED had [...] than IBS patients without ED" Appropriate translation and validation method.

Fairburn & Rothwell (2015)[19]	Systematic Clinical Review	To identify, characterize, and evaluate the clinical utility of apps designed either for people with EDs or for ED professionals.	One of the apps identified used the SCOFF as a screening measure however no further details on how the measure is used.	Unknown	Appropriate		Simply reporting on apps that utilise the measure.
Fragkos & Frangos (2013)[20]	Cross-sectional study	To assess factors predicting ED risk in a sample of undergraduate students.	SCOFF used as part of the self-report questionnaires. SCOFF used to screen for ED risk. A score of 2 or more was interpreted as someone being at risk for EDs	Original	Not appropriate	A	The SCOFF has not been validated to assess a person's *risk* of developing an ED.

Kroplewski et al. (2019)[21]	Cross-sectional study	To measure whether people at increased risk for EDs and people without an increased risk of EDs differ from each other in the assessment of family functioning and self-esteem dimensions	SCOFF used to determine individuals with an increased risk of EDs. Respondents who scored 2 or more on SCOFF were classified as being at high risk of developing an ED. The authors separated participants into two group – people who were low risk (less than two) and high risk (scoring more than 2).	Original	Not appropriate	A	The SCOFF has not been validated to assess a person's *risk* of developing an ED.
Sharifian et al. (2021)[22]	Cross-sectional survey	To investigate the association between dental fear and EDs through body mass index (BMI), and SCOFF	Used the SCOFF questionnaire to assess those at risk for developing EDs. Put participants into either 'positive SCOFF' or 'negative SCOFF'. Considered two or more "Yes" answers a positive result (SCOFF positive), suggesting a high probability of having an ED.<br>Used this 'SCOFF status' to associate with other variables.	Finnish	Not appropriate	A E	The SCOFF has not been validated to assess a person's *risk* of developing an ED. Description of the SCOFF in the methods and interpretation of the results was appropriate. However, their hypothesis indicates they were investigating 'risk of EDs'.
Ganson et al. (2021)[23]	Cross-sectional study	To estimate the associations between a positive ED screen and any lifetime ED diagnosis and illicit drug use among a large, diverse sample of college students.	Looked at association between a positive ED screen (measured using the SCOFF) and any self-reported lifetime ED diagnosis and self-reported illicit drug use in the past 30 days. Used a cut off score of 2 or more.	Original	Appropriate		Appropriate interpretation of the results as an 'ED screen'

Jacob. Haro & Koyanag (2018)[24]	Cross-sectional survey	To assess the association between ADHD symptoms and DE.	Used data from 2007 Adult Psychiatric Morbidity Survey (APMS). SCOFF scores included in the analyses and were used to identify 'possible ED'. In addition, analysed the 5 items on the SCOFF separately to assess ED symptoms.	Original	Not appropriate	E<br>D<br>C	Appropriate caution given when interpreting overall SCOFF scores (i.e., possible ED). However, analysing the SCOFF items separately is not a validated measure of ED symptoms.<br>Aim of the study states they are looking at DE. The SCOFF is not a valid measure of 'disordered eating', but of EDs.
Piacentino et al. (2017)[25]	Cross-sectional study	To evaluate the prevalence of body image disorders (BIDs) and EDs in PIED (performance and image enhancing drugs) using athletes vs. PIED nonusers.	The SCOFF was administered to people running well-known gyms and training centres over a 15-month period.<br>A score of >2 on the SCOFF indicated a probable ED, primarily AN or BN, or BDD (body dysmorphic disorder)	Italian	Not appropriate	I	The SCOFF has not been validated to screen for BDD.
Veses et al. (2015)[26]	Cross-sectional survey	To examine the association between cognition measurements and the risk of developing ED in adolescents considering their weight status.	The risk of developing EDs was measured using the SCOFF.	Original	Not appropriate	A	The SCOFF has not been validated to assess a person's *risk* of developing an ED.
Moser et al. (2020)[27]	Translation	To detail the translation and adaptation of SCOFF into Brazilian Portuguese	Two independent groups translated the original scale. The translation was then administered to a focal group of patients with ED. Then, a preliminary version of the scale in Brazilian Portuguese was sent for backtranslation by two English native speakers, who worked independently. The translation was sent to the original authors for approval. The authors describe the SCOFF as a brief ED screen.	Brazil-Portuguese	Mixed	H	Robust translation method, however, no validation of the adapted measure.

Shaikh & Kayani A. (2014)[28]	Cross-sectional	To determine the opinions and behaviours	The SCOFF was used in conjunction with a pre-tested questionnaire with close-ended	Original	Appropriate		Provides a level of caution with the results
		pertaining to dieting and body size among 16 to 20 year old female school and college students in Islamabad, Pakistan.	questions to detect EDs and study beliefs/behaviours about dieting and weight. A score of 2 or more on the SCOFF was indicative of a 'likely ED'				
Garcia et al. (2011)[29]	Validation	To evaluate the validity of the French version (SCOFF-F) of the SCOFF questionnaire for the detection of EDs among a female patient population referred to a clinical nutrition unit.	All participants completed the SCOFF then were assessed by an ED specialist (who was blind to their SCOFF result), who administered the MINI and then an interview according to DSM-5 criteria. Weight and height also measured	French	Appropriate		Appropriate use and method. The discussion is somewhat inappropriate as the authors suggest the SCOFF could be used to facilitate ED diagnosis, however they do not expand on what that would look like (i.e., initial screening followed by a robust diagnostic test).
Falvey et al. (2021)[30]	Cross-sectional study	To examine the association between military affiliation and ED symptoms among college students, and the likelihood of ED diagnosis among those with ED symptoms.	ED symptoms were measured using the 5item SCOFF questionnaire. Individuals who scored ≥2 on the SCOFF were classified as having symptoms of an ED.	Original	Not appropriate	B	Interpretation of results not cautious enough, not suggestive of a screen.
Bou Khalil et al. (2018)[31]	Cross-sectional study	To assess the level of disgust and fear feelings in situations generating avoidance in individuals who are at high risk of developing an ED.	The SCOFF questionnaire was used to assess the risk of developing an ED. A score of 2 or more indicated a higher vulnerability of developing an ED	Original	Not appropriate	A	The SCOFF has not been validated to assess a person's *risk* of developing an ED.

Elran-Barak et al. (2020)[32]	Cross-sectional survey	Used data of middle and high-school children in Israel in order to identify rates of DE among Arabs and Jews, while examining the relations of home family dinners (vs. other dinner options) with DE.	The SCOFF questionnaire was used to assess DE. A score of > 2 was considered a likely case of DE.		Not appropriate	C	The SCOFF is not a valid measure of 'disordered eating', but of EDs.
Hansson, Daukantaité & Johnsson (2017)[33]		To explore whether mothers' and fathers' own DE and emotion dysregulation, were associated with their daughters' or sons' DE and emotion dysregulation	Used back-translated version of SCOFF in Swedish. SCOFF used to assess DE. A threshold of 2 positive answers was proposed to indicate the probability of an existing ED, authors suggest that a score of 1 could indicate DE (unclear which cut-off the authors used in this study).	Swedish	Not appropriate	C	The SCOFF is not a valid measure of 'disordered eating', but of EDs.
Bachle, Stahl-Pehe & Rosenbauer (2016)[34]	Prevalence study	To estimate the prevalence of DE and insulin restriction (IR) among youths aged 11–21 years with early-onset type 1 diabetes (T1D) and a disease duration of at least 10 years.	All respondents answered the SCOFF questionnaire and reported on the frequency of IR and clinical outcomes. Screening for DE was positive when more than two SCOFF items were answered affirmatively.	German	Not appropriate	C	The SCOFF is not a valid measure of 'DE', but of EDs.
Saleh et al. (2018)[35]	Prevalence study	To examine the prevalence of DE attitudes among female students at An-Najah National University, Palestine	SCOFF described as a 'highly reliable and valid scale and used to assess the risk of EDs'. SCOFF used to measure 'DE attitudes'. A total score of ≥ 2 indicates that the participant has a high possibility of having AN or BN. In the discussion section, the authors acknowledge the limitations of the SCOFF questionnaire being a screening measure and not diagnostic.	Arabic	Not appropriate	C	The SCOFF is not a valid measure of 'disordered eating', but of EDs.<br>However, there was acknowledgement of the screening nature of the SCOFF tool.

Petisco-Rodríguez et al. (2020)[36]	Cross-sectional study	To investigate whether female athletes, particularly gymnasts and footballers, exhibit more eating problems compared to female nonathletes, and whether some factors are possible contributors to ED risk.	States that two positive answers in the five SCOFF questions indicate DE behaviour and attitudes. Authors do report that the SCOFF is "simple and easy to apply and score and is designed to raise suspicion of a likely case rather than a diagnosis"	Original	Not appropriate	C	The SCOFF is not a valid measure of 'disordered eating', but of EDs. However, there was acknowledgement of the screening nature of the SCOFF tool.

Ko et al. (2015)[37]	Cross-sectional study	To examine DE behaviours in university students in Vietnam.	Having two or more 'yes' responses on the SCOFF questionnaire indicates that the participant could have a high possibility of having AN or BN. In the discussion, interprets the SCOFF results as indicating participants had a "high possibility of having ED symptoms"	Vietnamese	Not appropriate	C D	The SCOFF is not a valid measure of 'disordered eating' or 'ED symptoms', but of EDs.
Ammann et al. (2018)[38]	Cross-sectional survey	To describe the characteristics of males at risk of DE while controlling for potential confounding factors.	Divided the surveyed males into two groups - positive SCOFF (score of 2 or more) and negative SCOFF (score of 1 or less). Described the SCOFF as "a validated screening tool for identifying subjects at-risk of DE"	French	Not appropriate	C	The SCOFF is not a valid measure of 'disordered eating', but of EDs.
Hazzard et al. (2020)[39]	Cross-sectional survey	To examine differences in ED risk and diagnosis by sexual orientation in a national sample of college students.	Analysed data from 2016-2019 Healthy Minds Study. ED risk was assessed with the SCOFF - a score of 2 or more indicated somebody at risk of an ED	Original	Not appropriate	A	The SCOFF has not been validated to assess a person's *risk* of developing an ED.

Calcaterra et al. (2020)[40]	Validation	To assess DE behaviours prevalence in youth with type 1 diabetes management, proposing new diagnostic subscales to represent the clinical dimensions associated with feeding and EDs	SCOFF used to validate a new measure of DEB in type 1 diabetes. Used a cut-off of >2 indicating a possible ED and the need of further evaluation.	Italian	Mixed	C	Does not appear to be using the SCOFF to assess disordered eating but validating an alternative measure.<br>However, as the SCOFF does not measure disordered eating it may not be the most appropriate tool to validate the new measure.
Vijayalakshmi et al. (2018)[41]	Cross-sectional study	To assess the presence of EDs among Medical and Nursing students	In the methods the authors indicate that "a score of two or more points indicates the patient may be suffering from an ED".<br>Interprets the results as participants "respond[ing] for DE behaviors", "be[ing] more vulnerable to EDs" and being "at risk for EDs"	Original	Not appropriate	E<br>C<br>A	Inconsistent interpretation of the SCOFF scores<br>The SCOFF is not a valid measure of 'disordered eating', but of EDs.<br>The SCOFF has not been validated to assess a person's *risk* of developing an ED.
Feng & Abebe (2017)[42]	Cross-sectional survey	To examine the prevalence and associated factors of DE behaviours among rural Chinese adolescents.	Describes the SCOFF as "an effective tool that can screen DEB quickly". Used the SCOFF to screen the prevalence of a "likely case of DE behaviour"	Chinese	Not appropriate	C	The SCOFF is not a valid measure of 'disordered eating', but of EDs.
Giel et al. (2013)[43]	Longitudinal	To identify the prevalence of ED symptoms in obese adolescents participating in a lifestyle intervention for weight loss, and to investigate possible relationships with weight change, general psychopathology, and health-related quality of life	Describes the SCOFF as a "self-report screening instrument assessing core symptoms of EDs".<br>In the results, the authors do use the phrase "screened positive for an ED", however also split the 5 items on the SCOFF up for independent analysis of 'ED symptoms'.	German	Not appropriate	D	Whilst this study appropriately reports the results of the SCOFF as a screen, analysing the SCOFF items separately is not a validated measure of ED symptoms

Burke et al. (2021)[44]	Cross-sectional survey	To examine prevalence estimates of ED pathology across several distinct multiracial groups.	Used the SCOFF to estimate prevalence of ED pathology. A score of 2 or more interpreted as a positive screen (i.e., likely ED diagnosis)	Original	Appropriate		Appropriately reports the results of the SCOFF as a screen. However, more caution in the interpretation of the results (particularly in the discussion) would have been beneficial.
Eisenberg et al. (2011)[45]	Longitudinal	To examine the prevalence, correlates, persistence, and treatment-seeking related to symptoms of EDs in a random sample of college students.	Measured the prevalence of ED symptoms using the SCOFF. Interprets results as a 'positive screen'. Adopted a threshold of 3 or more symptoms for a positive screen. Within the limitations, highlights that the SCOFF is a screening tool and not to be used for clinical diagnosis.	US	Mixed	F	Appropriate level of caution and interpretation of the results as a screen. However, no clinical rationale given for the altered scoring threshold
Tavolacci, Déchelotte & Ladner (2020)[46]	Cross-sectional study	To identify the characteristics of broader categories of EDs and help-and-care-seeking among college students.	Diagnostic threshold set at 2 positive responses. Data obtained with SCOFF gave a proxy of actual ED. Used the ExpaliTM algorithmic tool (combining SCOFF and body mass index) to screen EDs into three diagnostic categories (restrictive ED, bulimic ED, hyperphagic ED and no ED)	French	Not appropriate	B	The SCOFF questionnaire is designed as a screening measure The categories of ED used in the Expali algorithm were derived from DSM-5, however the SCOFF validated in line with the DSM-IV criteria.
Azzouzi et al. (2019)[47]	Cross-sectional survey	To evaluate EDs among Moroccan medical students with a focus on	The SCOFF was included in the questionnaire. Stated that two positive responses are highly predictive of EDs.	French	Not appropriate	A	The SCOFF has not been validated to assess a person's *risk* of developing an ED
		cognition and behaviour related to EDs	Subjects with a positive SCOFF score were defined as being 'at risk of EDs'.				

Tavolacci et al. (2015)[48]	Cross-sectional study	To determine the prevalence of EDs among university students and its relationship to behavioural characteristics and substance use	The SCOFF is used in the online questionnaire to assess prevalence of ED risk. Used a cut-off point of 2 positive answers. In the results section, the SCOFF scores are labelled 'positive and negative SCOFF scores'. In the discussion, interprets the SCOFF scores as 'risk of ED'.	French	Not appropriate	A	The SCOFF has not been validated to assess a person's *risk* of developing an ED. Some appropriate wording when reporting the SCOFF results (i.e., 'positive SCOFF score')
Phillips et al. (2015)[49]	Cross-sectional	To examine associations between spirituality and EDs in a sample of college students	The SCOFF was used in a paper questionnaire pack given to nursing students. Uses a cut-off of 2 positive answers to indicate a 'likely case of an ED'. The results are interpreted as 'positive SCOFF score' indicating 'a need for a more detailed evaluation by a mental health professional'. Similar interpretations in the discussion section.	Original	Appropriate		Appropriate level of caution when interpreting the results. Highlights the need for further professional evaluation before diagnosis given.
Damiri et al. (2021)[50]	Cross-sectional	To investigate the prevalence and the association between ED and cognitive enhancers, and psychostimulants use among An-Najah National University students.	The SCOFF was included as part of the survey completed. Uses a score of 2 or more to indicate a likely diagnosis of AN or BN. Reports the SCOFF scores as demonstrating the 'prevalence of EDs'	Arabic	Not appropriate	B	Insufficient caution when interpreting the results. The SCOFF, as a screening tool, is not able to measure the prevalence of EDs.
Memon et al. (2012)[51]	Cross-sectional	To find out the frequency of EDs among medical students of Karachi and design strategies to overcome them.	Data was collected using the SCOFF (among other questionnaires). Used a cut of point of more than 2 positive answers. Results interpreted as participants being at 'high risk of EDs'.	Original	Not appropriate	A	The SCOFF has not been validated to assess a person's *risk* of developing an ED.
Fursland & Watson (2014)[52]	Cross-sectional	To identify the prevalence of eating problems, using the	All participants were administered the SCOFF. If scored >2, then they were administered a standardised diagnostic	Original	Appropriate		Appropriate level of caution given to the interpretation of

		SCOFF, and EDs when screening positive on the SCOFF (i.e., ≥2), among patients seeking help for anxiety and depression at a community-based mental health service.	interview (MINI) by a Clinical Psychologist. Interpreted positive screens on the SCOFF as 'eating problems' and only gave a diagnosis of 'ED' following the diagnostic interview.				SCOFF scores and robust diagnostic procedure followed.
Safer et al. (2020)[53]	Cross-sectional	To assess prevalence and associated factors of EDs among health occupation students in the university of Monastir.	The SCOFF was completed anonymously by university students. It was used to assess the prevalence of ED and risk factors associated with a positive score. A score equal or greater than two indicated a 'likely case of ED'. In the results section, the authors referred to the separate items on the SCOFF as different 'disorders'. Interpreted positive SCOFF screens as assessing 'prevalence of EDs'.	French	Not appropriate	B	Insufficient caution used when interpreting the results. As a screening tool, the SCOFF cannot be used to measure prevalence of EDs.
Štefanová, Bakalár & Baška (2020)[54]	Cross-sectional	To explore the prevalence of eatingdisordered behaviour in adolescents, and explore its associations with body image, body composition and physical activity in this age group.	Data obtained from the World Health Organization collaborative health behaviour in school-aged children (HBSC) study conducted in 2018. SCOFF included in that data to explore eating-disordered behaviour. A positive screening status was defined as ≥2 positive answers". Positive SCOFF screens interpreted as 'eating-disordered behaviour'.	Original (although stone changed to kg for 'O' item due to language differences)	Not appropriate	C	The SCOFF screens for possible cases of EDs, and not for eatingdisordered behaviour.
Berger et al. (2014)[55]	Quasiexperimental pre–post	To assess the effectiveness of a German school-based primary prevention program for eating behaviours ("Torera") for seventh graders	Eating behaviour was assessed using the SCOFF (and EAT). Reported that a score of ≥2 indicates a 'likely case of anorexia nervosa or bulimia'.<br>SCOFF was used to indicate changes in eating behaviour and risky eating behaviour following engagement in the Torera program.	Original	Not appropriate	C	The SCOFF screens for possible cases of EDs, and not for eating behaviour.

Hosoda (2021)[56]	Validation	This study aimed to validate the SCOFF questionnaire in relation to the Eating Disorder Examination Questionnaire (EDE-Q) and to examine the appropriateness of a question concerning weight loss among Japanese university students.	SCOFF was included in a questionnaire administered to university students. The original SCOFF questionnaire was translated into Japanese by four experts in EDs, back-translated into English, and subsequently approved by the original author. To examine test–retest reliability, SCOFF was re-administered at two- to four-week intervals. The authors also assessed internal consistency and validity of the Japanese SCOFF.	Japanese	Appropriate		Appropriate translation and validation method.
Sánchez-Armass et al. (2012)[57]	Cross-sectional	To examine the psychometric performance of the SCOFF among Mexican university students.	SCOFF administered to participants and the psychometric properties of examined. Scores above 2 considered to be a 'positive screen for AN or BN'. In the results, the authors interpret a positive screen as being 'at risk of an ED'.	Spanish	Mixed	A	Appropriate exploration of psychometric properties. However positive SCOFF screens were inappropriately interpreted as demonstrating 'risk of ED' - the SCOFF has not been validated to assess a person's *risk* of developing an ED.
Lichtenstein et al. (2018)[58]	Cross-sectional	To develop and test the psychometric properties of a youth version of the Exercise Addiction Inventory (EAI).	SCOFF was used as part of the questionnaire administered to participants. Used to estimate the probability of ED pathology. Used a cut of point of 2 or more to indicate 'risk of ED'. SCOFF was also used to represent separate ED symptoms in a logistic regression. In the results and discussion section the SCOFF is used to identify participants in the 'patients with EDs group'	Original	Not appropriate	B A	Insufficient caution used when interpreting the results (i.e., 'patients with EDs'). In addition, SCOFF at times used to indicate someone being 'at risk of ED' - the SCOFF has not been validated to assess a person's *risk* of developing an ED.

Prnjak et al. (2020)[59]	Cross-sectional	To determine a threshold score for the Eating Disorder Examination – Questionnaire Short (EDE-QS) that could be used in screening for probable ED cases in community settings	SCOFF, among other questionnaires, was used to determine appropriate cut-off scores for the EDE-QS.<br>In the methods section the authors do state that a score of ≥ 2 is 'indicative of an ED'	Original	Appropriate		Appropriate use of the SCOFF as a screening tool.<br>However, insufficient caution used in the description of the tool (i.e., 'indicative of an ED). This does not impact the use of the tool in this study, however.
Vijayalakshmi (2017)[60]	Cross-sectional	To assess gender disparity in body weight perception, BMI, weight satisfaction and role of depression among	Students completed the SCOFF amongst other questionnaires. In the methods the authors state that a score of two or more points 'indicates the patient may be suffering from an eating disorder'. Interprets scores as	Original	Not appropriate	A C	The SCOFF has not been validated to assess a person's *risk* of developing an ED. The SCOFF is not a valid measure of 'disordered eating', but of EDs.
		undergraduate Medical and Nursing students.	scoring 'positively for DE behaviours' and being at 'high risk for development of EDs'.				
Forbush, Wildes & Hunt (2013)[61]	Cross-sectional	To present normative and psychometric data for the Eating Pathology Symptoms Inventory (EPSI) in a large sample of college men and women	SCOFF used to measure reliability and validity of EPSI.	Original	Appropriate		Appropriate use of the SCOFF questionnaire as a screening tool
Czeglédi & Szabo (2016)[62]	Cross-sectional	To explore the psychometric properties of the Beliefs About Attractiveness Scale - Revised (BAA-R) and its correlates among young adults in Hungary.	Used the SCOFF to explore correlates between risk of EDs and scores on BAA-R. States that a score of 2 or more 'indicates suspicion of AN or BN'. In the results the authors interpret the scores as participants being 'at risk of developing an ED'	Hungarian	Not Appropriate	A E	Appropriate description of the SCOFF in the methods but inappropriate interpretation of the scores in the results and discussion. The SCOFF has not been validated to assess a person's *risk* of developing an ED.

Lichtenstein, Hemmingsen & Støving RK (2017)[63]	Validation	To test the psychometric properties of the SCOFF in adolescents aged 11–20 years and for the first time validate the questionnaire in Danish.	Tested psychometric properties of the Danish version of SCOFF. Translated and back translated. Assessed internal consistency, specificity, sensitivity and construct validity.	Danish	Appropriate		Appropriate translation and validation method. Appropriate description of the SCOFF.
Bénard et al. (2019)[64]	Cross-sectional	To explore the relation between impulsivity, dietary intake, and EDs in a general population. To assess whether impulsivity was associated with energy intake, food-group consumption, snacking, and risk of EDs.	SCOFF used to assess risk of EDs. Stated that a score ≥2 indicates 'risk of ED'. In addition, uses the Expali algorithm in order to allow the distinction between ED categories	French	Not appropriate	A B	The SCOFF has not been validated to assess a person's *risk* of developing an ED. The SCOFF questionnaire is designed as a screening measure. The categories of ED used in the Expali algorithm were derived from DSM-5, however the SCOFF validated in line with the DSM-IV criteria.
Wróblewska et al. (2018)[65]	Longitudinal	To evaluate the impact of biopsychosocial factors involved in food allergy on the prevalence of EDs.	The SCOFF was used to assess the frequency/prevalence of EDs (alongside the reference EAT-8). States that two or more affirmative answers is 'indicative of an ED'.	Original	Not appropriate	B	Insufficient caution used. The SCOFF is unable to assess the prevalence of EDs due to its screening nature.
Baechle et al. (2014)[66]	Cross-sectional	In this study, the prevalence of disordered eating behaviour (DEB) in intensely treated children and adolescents with early-onset Type 1 diabetes and peers from the general population was compared.	The SCOFF was used as a screening instrument to identify participants with symptoms of DEB. Removed question 5 in some analyses due to it potentially resulting in an overestimation of SCOFF positive in those with diabetes. In addition, the items on the SCOFF were separated for some analyses.	German	Not appropriate	C D	The SCOFF is not a valid measure of 'disordered eating behaviour', but of EDs. Analysing the SCOFF items separately is not a validated measure of ED symptoms

Hicks et al. (2013)[67]	Cross-sectional study	To look at a cohort of female adolescent patients to ascertain how much they knew about EDs, if they had practised behaviour consistent with BN and/or AN, and if there was a disconnect with actual and perceived healthy weight status.	SCOFF used to examine whether the participant practises a behaviour consistent with an ED. A score of two or more indicated an ED may exist. Results interpreted as 'people with a possible ED'. Also analysed the individual items to explore ED behaviours. Split participants into 'ED group' and non-ED group', occasionally used the term 'suspected'.	Original	Mixed	E<br>D	At times an appropriate level of caution was used (i.e., 'possible ED', 'suspected ED') however this was not used consistently. In addition, analysing the SCOFF items separately is not a validated measure of ED symptoms or behaviours
Esteban-Gonzalo et al. (2014)[68]	Cross-sectional	To compare the risk of having an ED among immigrant and native adolescents living in Madrid and to determine the possible influence of length of residence on the risk of the immigrants.	Having a risk of ED, was estimated according to the SCOFF. Two or more positive answers to the questions were taken to indicate being at risk of an ED.	Spanish	Not appropriate	A	The SCOFF has not been validated to assess a person's *risk* of developing an ED.
Muros et al. (2020)[69]	Cross-sectional	To describe and predict EDs according to sex, body mass index, age and sport discipline within a sample of athletes.	Method states SCOFF was used to assess DEB. ≥2 was considered as a positive indication of an ED. In the results section the SCOFF scores are interpretated as assessing participants 'at risk of ED'.	Spanish	Not appropriate	E<br>C<br>A	Inconsistent description and interpretation of the SCOFF The SCOFF is not a valid measure of 'disordered eating behaviour', but of EDs.
							The SCOFF has not been validated to assess a person's *risk* of developing an ED.
Spillebout et al. (2019)[70]	Cross-sectional	To investigate the coexistence of ED and IBS among university students in France.	The SCOFF was used in order to assess eating behaviours. A score of 2 or more was used to indicate a possible ED. Interpreted a positive screen on the SCOFF as a participant having an ED, measuring 'prevalence of ED'.	French	Not appropriate	B	The SCOFF is unable to assess the prevalence of EDs due to its screening nature.

Querol et al. (2016)[71]	Cross-sectional	To evaluate whether adolescents with a positive score in a screening tool for possible EDs have a different diet intake in comparison with those with a negative score.	The number of possible cases of ED was assessed by the SCOFF. A score of >2 indicated a 'likely case of AN or BN'. In this study, all participants who scored more than 2 positive answers were considered as being positive in SCOFF, while the rest were coded as SCOFF negative.	Spanish	Appropriate		An appropriate level of caution is used when interpreting the results. The authors are clear on the SCOFF's role as a screening tool as opposed to being diagnostic.
Jugale et al. (2014)[72]	Cross-sectional	To detect the presence of EDs and the clinical manifestations in 20-25 year old women in Bangalore city.	SCOFF was used to screen for suspected cases of AN and BN. Participants who scored more than 2 (although in the methods section states more than 1) on the SCOFF were suspected AN or BN cases.	Original (although changed reference to stone to kg in question 3)	Appropriate		An appropriate level of caution is used when interpreting the results. The authors are clear on the SCOFF's role as a screening tool as opposed to being diagnostic.<br>However, inconsistencies in what the threshold was for positive screen.
Veses et al. (2014)[73]	Cross-sectional	To analyse the individual and combined influence of overweight and physical fitness on the risk of developing EDs in Spanish adolescents.	The risk of EDs was evaluated using the SCOFF questionnaire. A score of two or more on the SCOFF was interpreted as being 'at risk of an ED'.	Original	Not appropriate	A	The SCOFF has not been validated to assess a person's *risk* of developing an ED.
Solmi et al. (2014)[74]	Cross-sectional	To estimate the prevalence and correlates of DE in a general population sample based in South East London.	DE was assessed using the SCOFF questionnaire. Also analysed the individual items when exploring associations of DE with other factors.	Original	Not appropriate	C D	The SCOFF is not a valid measure of 'disordered eating', but of EDs.<br>Analysing the SCOFF items separately is not a validated measure of ED behaviours.

Zeiler et al. (2016)[75]	Cross-sectional	To investigate the prevalence of ED risk as well as associated psychopathology and health-related quality of life in a large population sample of Austrian adolescents.	The SCOFF questionnaire was used to identify participants at risk for EDs. Individuals were defined at risk for EDs if they confirmed at least two of the five items.	German	Not appropriate	C	The SCOFF is not a valid measure of 'disordered eating', but of EDs.
Bizri et al. (2020)[76]	Cross-sectional	To evaluate the prevalence and possible factors associated with EDs among medical students at the American University of Beirut in Lebanon.	The SCOFF (alongside the Eating Attitudes Test-26 questionnaire) was used to assess ED risk. A total score of 2 or more on the SCOFF was deemed 'high-risk'.	Original	Not appropriate	A	The SCOFF has not been validated to assess a person's *risk* of developing an ED.
Conceicao et al. (2017)[77]	Two-stage epidemiological study	To examine the point prevalence of EDs and picking/nibbling in elderly women.	The SCOFF was included in the screening stage of the study.<br>Participants progressed to the assessment stage if they scored at least one positive item on the SCOFF and at least one positive answer on the picking/nibbling or night eating syndrome screening questions.<br>At the assessment stage participants were interviewed by a Psychologist using the EDs Examination interview to establish a diagnosis	Original	Mixed	F	Appropriate use of the SCOFF as a screening tool, with further clinical assessment to establish diagnosis.<br>However, no clinical rationale given as to why threshold score changed.

McBride et al. (2013)[78]	Cross-sectional	This study examined the prevalence and nature of DE patterns in the National Adult Psychiatric Morbidity Survey 2007.	A score of 2+ on the SCOFF was interpreted as a possible eating disorder which would warrant further clinical assessment.<br>Responses to the SCOFF and body mass index (BMI) were analysed using latent class analysis to establish DE patterns.<br>The ordering of the SCOFF questions was slightly re-arranged so that the subject context (i.e., DE) became clear and also to reduce the shock that a respondent might feel about being asked about induced vomiting first.	Original (with amendment)	Not appropriate	B	Appropriate description of the SCOFF as a screening tool initially<br>However, use the SCOFF to establish sub-types of eating disorders and explore their prevalence
Koyanagi, Stickley & Haro (2016)[79]	Cross-sectional	To assess the association of subclinical psychosis with ED symptoms and possible EDs using nationally representative population-based data from the 2007 Adult Psychiatric Morbidity Survey (APMS) conducted in England.	The SCOFF was used to assess possible ED. An affirmative reply to at least two items was considered a positive screen	Original	Appropriate		Appropriate level of caution used when interpreting the results. Identified the SCOFF as a screening tool and not diagnostic.
Pustivšek, Hadžic & Dervišević E (2014)[80]	Cross-sectional	To examine the risk factors and the prevalence of risk for ED among male adolescent elite athletes and nonathletic controls.	Risk for ED was determined using the SCOFF.	Slovenian	Not appropriate	A	The SCOFF has not been validated to assess a person's *risk* of developing an ED.

Pustivšek et al. (2019)[81]	Cross-sectional	To investigate the risk for EDs and compare body composition in participants with and without risk in adolescent Slovenian athletes and aged matched controls.	Risk for ED was determined using the SCOFF	Slovenian	Not appropriate	A	The SCOFF has not been validated to assess a person's *risk* of developing an ED.
D'Anna et al. (2021)[82]	Cross-sectional	To provide the prevalence of individuals at risk for ED psychopathology in a representative population of Italian adolescents aged 14–19.	The risk for EDs was assessed through the SCOFF. A cut-of score of 3 was used for 'risk of an ED'.	Italian	Not appropriate	A<br>F<br>D	The SCOFF has not been validated to assess a person's *risk* of developing an ED. No clinical rationale given for altered cut-off score.<br>The SCOFF should be used as a collective screening measure and not to indicate separate ED symptoms.
Hansson, Daukantait & Johnsson (2015)[83]	Validation	To examine the validity of the SCOFF in a Swedish sample using the EDE-Q as a "reference standard".	The SCOFF was used to assess DE behaviour. Original questionnaire translated into Swedish using the back translation method.	Swedish	Not appropriate	C	The SCOFF is not a valid measure of 'disordered eating behaviour', but of EDs. The EDE-Q would not be appropriate to validate the SCOFFs use as a screening tool for DEB as it too assesses EDs as opposed to DE.

Yao et al. (2020)[84]	Cross-sectional	To present prevalence and sociodemographic factors associated with screen-detected DE and related traits in a population-based sample of women in China.	Used data from the China Health and Nutrition Survey (CHNS) in 2015. 'Screendetected DE' was defined as endorsing two or more of the five SCOFF items. Developed algorithms to identify patterns of SCOFF responses reflecting clinical presentations that were more typical of AN, BN, and BED.	Chinese	Not appropriate	C	The SCOFF is not a valid measure of 'disordered eating', but of EDs.
Richter et al. (2017)[85]	Validation	To evaluate whether the SCOFF can be used as a screening tool for DE in universal samples.	Uses the SCOFF to screen for DE. References previous studies that have used the SCOFF in this way, however each of these studies either has not validated the SCOFF for screening DE or has not screened for DE.<br>Uses a subscale of the ICD-10-SymptomRating questionnaire (ISR-E) as a reference criteria however this scale assesses EDs, not DE.	German	Not appropriate	C	The SCOFF is not a valid measure of 'disordered eating', but of EDs.
Tavolacci et al. (2019)[86]	Cross-sectional observational	To evaluate the performance of a clinical algorithm to identify 4 broad categories of EDs.	The authors state that 'a positive SCOFF score indicates an ED with at least 2 positive answers to the 5 questions'. The authors developed a clinical algorithm combining at least two positive answers to the SCOFF test and one of the four BMI levels to assign participants to one of four broad categories of EDs.	French	Not appropriate	B	The SCOFF questionnaire is designed as a screening measure and, in addition, is validated in line with the DSM-IV criteria.
Kronfol et al. (2018)[87]	Cross-sectional	To assess the frequency of selected psychiatric problems among college students in two Arab countries: Qatar and Lebanon, and to compare them to the USA.	SCOFF was used to screen for EDs (although in results section the wording shows no level of caution - 'prevalence of EDs'). Used a cutoff of 3 or more positive answers, however no clinical rationale given as to this. No use of clinician to officially diagnose ED.	Original	Not appropriate	B F	Insufficient level of caution when interpreting the results, due to its screening nature the SCOFF is unable to assess prevalence of EDs.<br>No clinical rationale given for altered cut-off score.

Liu et al. (2015)[88]	Validation	To examine the comparative validity of the SCOFF questionnaire for detecting ED cases in men and women in the psychiatric outpatient setting	SCOFF was translated into Mandarin from the Chinese version via a back translation method.<br>Participants completed the SCOFF questionnaire and were interviewed using the ED Module of the Structured Clinical Interview for the DSM-IV-TR.<br>To assess test–retest reliability, a subsection of participants were asked to repeat the SCOFF questionnaire 7 to 14 days after the first assessment.<br>Reported that A score of ≥2 on the SCOFF indicated a 'likely ED case'	Mandarin Chinese	Appropriate		Appropriate level of caution when interpreting the results. Further clinical assessment completed before diagnosis made.<br>Appropriate translation method.
Andreeva et al. (2019)[89]	Cross-sectional	To expand knowledge about ED by analysing their presence and sociodemographic correlates in a very large sample of European adults recruited from the general population	Uses the SCOFF and the Expali algorithmic tool to assess EDs in the French general population.	Original	Not appropriate	B	The SCOFF questionnaire is designed as a screening measure. The categories of ED used in the Expali algorithm were derived from DSM-5, however the SCOFF validated in line with the DSM-IV criteria.
Barry, Sonneville & Leung (2021)[90]	Cross-sectional	To explore the relation between food insecurity and screening positive for an ED among students attending a large, public Midwestern university	A score of 2 or more on SCOFF was interpreted as 'screening positive for an ED' or 'positive SCOFF screen'<br>The language of the original questionnaire was modified slightly for use in a Midwestern population by substituting the words 15 pounds for the British 1 stone in Question 3.	Original	Mixed	E	Appropriate identification of the results as a screen, however a more consistent level of caution would be preferred (e.g., screen positive for a *possible* ED)
Bächle et al. (2015)[91]	Cross-sectional	To analyse the prevalence of and association between symptoms of EDs and depression in female and male emerging adults with early-onset,	The SCOFF questionnaire was used to screen for eating problems. If two or more questions were answered positively, then an ED was suspected. Reports the results as 'screening positive for an ED'. Also interprets results as participants with or without ED symptoms.	Original	Not appropriate	B D	More caution needed when interpreting the results (e.g., screen positive for a *possible* ED).<br>The SCOFF is not a measure of specific ED symptoms, but of EDs as a whole.

		longduration type 1 diabetes.					

Zarychta, Luszczynska & Scholz (2014)[92]	Longitudinal	To test the relationship between automatic thoughts about eating and the actual-ideal weight discrepancies, and their role in the formation and maintenance of ED symptoms in a nonclinical sample of adolescents.	SCOFF was used as a screening tool to assess ED symptoms. Results were interpreted as demonstrating 'higher levels of ED symptoms'.<br>Translation of SCOFF to Polish was conducted using the decentering technique.	Polish	Not appropriate	D	The SCOFF is not a measure of specific ED symptoms, but of EDs as a whole.
Teixeira et al. (2021)[93]	Translation & validation	To translate, culturally adapt, and validate a Brazilian version of the SCOFF questionnaire in a population of young adults.	Used the back translation method. The study calculated the sensitivity, specificity, and positive and negative predictive values for each test's threshold with a 95% confidence interval. Used the structural interview with the MINI to assess the validity of the SCOFF as a screening tool for EDs.	Brazilian Portuguese	Appropriate		Appropriate translation and validation method.<br>Ideally, some more caution would be given in the interpretation of SCOFF results<br>- e.g., 'screening for possible EDs'

Philipp et al. (2014)[94]	Cross-sectional	Pilot study conducted as part of the project risk management of the MHAT-Study to improve the main study. Aimed to determine the acceptability and feasibility of the screening phase, to estimate the prevalence of mental health problems and to perform a Non-Responder Analysis.	The SCOFF was used to determine the signs of disturbed eating habits. Authors proposed an alternative cut off criteria based on the clinical relevance of the SCOFF items - a score of 2 or more which had to include either item 1 (vomiting) or item 2 (weight reduction), as these are 'stronger indications of an ED'. Original SCOFF criteria (>2) resulted in 20.8% being screened for ED, with implemented criteria (one of the two items), this dropped to 6.6%.	German	Not appropriate	C F	The SCOFF is designed to screen for EDs, as opposed to 'disturbed eating habits'. Insufficient clinical rationale provided for the altered cut-off threshold.
Dooley-Hash et al. (2012)[95]	Cross-sectional	To describe the rates of EDs in patients aged 1420 who present to the emergency department for any reason and examines the relationship between EDs, depression, and substance use in these patients.	Modified version of SCOFF used to reflect younger age, linguistic differences between US and UK and to include BED and EDNOS. Modifications detailed in the paper. SCOFF results interpreted as 'screening positive for an ED', 'presence of an ED' and 'participants with an ED'	Modified version of original	Not appropriate	E	Inconsistent caution used when interpreting the results.
Dooley-Hash et al. (2019)[96]	Cross-sectional	To describes the prevalence of EDs among adult patients who present to the emergency department for medical care and examines the relationship between EDs,	Modified version of SCOFF used to reflect linguistic differences between US and UK and to include BED and EDNOS. Modifications detailed in the paper. SCOFF results interpreted as 'screening positive for an ED'. In the discussion section the authors report the 'prevalence of EDs' from the study.	Modified version of the original	Not appropriate	B	Clear distinction of the results as a screen, however more caution needed ideally - e.g., 'screened positive for a possible ED'. Due to its screening nature the SCOFF is unable to assess prevalence of EDs.

		depression, and substance use disorders.					
Aykut & Bilici (2021)[97]	Cross-sectional	To evaluate the relation between meal patterns and the risk of EDs in university students.	Used the SCOFF and EDE-Q to determine eating behaviour disorders, with face-to-face interviews by the researchers. Interprets SCOFF results as indicating 'risk of ED'	Original	Not appropriate	A	The SCOFF has not been validated to assess a person's *risk* of developing an ED.
Adelantado-Renau et al. (2018)[98]	Cross-sectional	To analyse the association between the risk of ED and academic performance in adolescents, considering the key role of weight status.	The SCOFF was used to measure 'risk of ED' - the higher the score, the higher the risk.	Spanish	Not appropriate	A	The SCOFF has not been validated to assess a person's *risk* of developing an ED.
Garrido-Miguel et al. (2019)[99]	Cross-sectional	To analyse the relationship between the risk of ED and bone health and to examine whether this relationship is mediated by body composition and cardiorespiratory fitness.	The SCOFF was used to measure 'risk of ED'. Scores were categorized into 2 groups - "risk of EDs" (≥2) and "no risk of EDs" (<2).	Spanish	Not appropriate	A	The SCOFF has not been validated to assess a person's *risk* of developing an ED.
Hautala et al. (2011)[100]	Prospective	To investigate the course of eating disturbance from middle to late adolescence, test certain	The SCOFF was used to identify ED symptoms in adolescents	Finnish	Not appropriate	D	The SCOFF is not a measure of specific ED symptoms, but of EDs as a whole.

		individual and parental factors and estimate the predictive power of these factors for the recurrence of eating disturbance and/or onset of new presentations.	Used a cut-off point of 1 positive score due to the population being adolescents, in line with previous research cited.				
Hansson, Daukantaitė & Johnsson (2016)[101]	Cross-sectional	To explore the relationships between four indicators of DE to identify typical DE pattern.	The SCOFF was used to measure DE. Split the SCOFF results into two 'categories of DE behaviour' (≥2 or ≤1 "yes" responses)	Original	Not appropriate	C	The SCOFF is not a valid measure of 'DE', but of EDs.
Lipson & Sonnville, (2020)[102]	Cross-sectional	To examine suicide risk by ED severity and symptom presentation in a nationwide sample of college students.	Analysed data from 2015-2017 Healthy Minds Study. The SCOFF was used to provide an ED symptom count (number of "yes" answers on the SCOFF, ranging from 0 to 5).	Original	Not appropriate	D	The SCOFF is not a measure of specific ED symptoms, but of EDs as a whole.
Aoun et al. (2015)[103]	Translation & Validation	To validate an Arabic version (A-SCOFF) of the SCOFF	SCOFF was translated into Arabic via back translation method. The validity of the ASCOFF was assessed by calculating the sensitivity and specificity for all possible screening cut-off points. The validated Arabic version of the MINI, together with DSM-IV criteria for eating disorders, were used as the gold standard for a diagnosis of eating disorder.	Arabic	Appropriate		Appropriate translation and validation method.
Solmi et al. (2015)[104]	Cross-sectional	To validate the SCOFF as a screening tool for ED in a multi-ethnic general population sample of adults.	Authors employed data from the South East London Community Health Study (SELCoH). Participants completed the SCOFF and a clinical assessment of ED using the ED section of the Structured Clinical Interview for DSM-IV.	Original	Appropriate		Appropriate validation method.

Sanchez-Armass (2017)[105]	Validation	To evaluate the criterion validity and diagnostic utility of the SCOFF in a Mexican sample.	Participants completed the SCOFF, and a random subsample of these completed the Eating Disorder Examination (EDE). The authors describe the study as 'detecting EDs in a Mexican university sample'. Some references to the SCOFF measuring 'DE'.	Original	Mixed	B<br>C	Appropriate validation method. Ideally more caution would be used when interpreting the results.<br>The SCOFF does not measure 'disordered eating' but EDs
Wan Wahida, Lai & Abdul Hadi (2017)[106]	Validation	To validate the SCOFF on a non-clinical sample of Malaysian students.	Measured content validity, convergent validity (using the EAT-26), internal consistency, test-retest reliability, specificity and sensitivity.	Original	Appropriate		Appropriate validation method
Di Lodovico, Dubertret & Ameller (2018)[107]	Cross-sectional	To assess risk of exercise addiction in runners at risk for EDs and to assess sociodemographic, behavioural and psychological characteristics distinguishing runners at-risk of and not-at-risk of EDs.	SCOFF used to assess ED risk. Participants scoring 2 or more were categorised as 'at risk of ED'.	French	Not appropriate	A	The SCOFF has not been validated to assess a person's *risk* of developing an ED.
Aoun et al. (2013)[108]	Cross-sectional	To observe whether the modification of eating behaviour related to stress during wartime increases the risk of developing an ED among civilians exposed to war stress during adulthood	SCOFF used to screen for EDs. A score of 2 or more was referred to as a 'positive SCOFF screening'. At times does refer to 'detection of ED' and 'ED risk' although this is minimal.	Original	Appropriate		Appropriate use of the SCOFF questionnaire as a screening tool, however more consistency in the reporting of the results would be ideal.

Cotton, Ball & Robinson (2003)[109]	Cross-sectional	To validate and compare the SCOFF with the Eating Disorder Screen for Primary Care (ESP).	Participants completed the Questionnaire for Eating Disorder Diagnoses (Q-EDD) which has been validated for diagnostic use, the SCOFF and the ESP. Sensitivity, specificity and likelihood ratios calculated. Performance of SCOFF and ESP was compared.	Original	Appropriate		Appropriate validation method.
Rodríguez et al. (2004)[110]	Cross-sectional	To analyse the differences in family functioning and socioeconomic status between subjects with disorders of eating behaviour and the healthy population	SCOFF used to exclude participants from the control group if they scored 2 or more and thus indicated a 'high probability of having disorders of eating behaviour'	Original	Mixed		Although the SCOFF generally should not be used to exclude participants from a control group (as it can include a high number of false positives), the authors of this study state that by scoring positive on the SCOFF a participant has a 'probability' of having an ED. A stronger level of caution should ideally be used.
Siervo et al. (2005)[111]	Validation	To explore the validity and reliability of the SCOFF, EAT 26 and TFEQ in a population of young women.	Analysed reliability, validity, sensitivity and specificity of the SCOFF. A psychiatrist assessed and diagnosed ED based on DSM-IV criteria. In the methods section authors state that a positive scoff screen indicates 'abnormal eating behaviour', however rest of interpretation appropriate.	Italian	Appropriate		Appropriate validation method (possible translation difference in use of "abnormal eating behaviour")

Rodríguez Martin et al. (2005)[112]	Case-control	To describe the personal and family antecedents and clinical characteristics of patients with EDs in a population of the south of Spain.	The SCOFF test was used to exclude subjects with high probability of EDs (a score of 2 or more)	Original	Mixed		Although the SCOFF generally should not be used to exclude participants from a control group (as it can include a high number of false positives), the authors of this study state that by scoring positive on the SCOFF a participant has a 'probability' of having an ED. A stronger level of caution should ideally be used.
Parker, Lyons & Bonner (2005)[113]	Cross-sectional	To assess the screening capability of the SCOFF	Assessed the screening capability of the SCOFF by comparing to the EDE-Q as the 'gold standard' for diagnosis (authors acknowledged the limitations of using the EDE-Q but felt to be the most appropriate method due to limited resources). A score of 2 or more indicated a 'potential ED' and/or 'at risk for an ED' (although risk is mentioned only once). In the discussion, the authors discuss the need for 'further evaluation' following a positive SCOFF screen.	Original	Appropriate		Appropriate methodology and mainly appropriate interpretation of the SCOFF, however ideally more caution would be used.
Farrow & Blissett (2005)[114]	Longitudinal	To investigate the relationship between maternal psychopathological symptomatology during pregnancy and at 6 and 12 months postnatally and maternal use of controlling and restrictive feeding practices at 1 year	SCOFF used to assess ED psychopathology. Participants were split into two groups (ED and No ED) depending on their SCOFF scores. Term 'possible ED' used occasionally.	Original	Not appropriate	B	Insufficient level of caution used when interpreting the results.

Garcia-Campayo et al. (2005)[115]	Translation and Validation	To assess the performance of the Spanish version of the SCOFF for the detection of EDs in primary care	Translated the SCOFF into Spanish via a back translation method.<br>Administered the SCOFF to Spanish patients to assess if the questionnaire was acceptable and understandable for them. Participants diagnosed with ED by psychiatrists using the Schedules for Clinical Assessment in Neuropsychiatry as a gold standard. Validity and reliability of the SCOFF assessed.	Spanish	Appropriate		Appropriate translation and validation method.
Noma et al. (2006)[116]	Cross-sectional	To examine the appropriateness of each question of the SCOFF in comparison with the Eating Attitudes Test-26	The SCOFF and the EAT-26 were administered to 80 patients with EDs who had received treatment in Japan over the course of 6 months. A score of 2 or more indicated someone likely had an ED<br>The SCOFF was first translated into Japanese by the authors, after which a bilingual Japanese psychiatrist assessed the accuracy of the Japanese version of the test<br>Overall SCOFF scores and scores from individual questions were correlated with the EAT-26 scores.	Japanese	Not appropriate	D<br>H	The SCOFF should be analysed as a single, categorical score. A more robust translation method could have been used and no validation of translated measure
Johnston et al. (2007)[117]	Cross-sectional	To assess the feasibility of screening for DE within primary care	The SCOFF questionnaire was used to screen for DE.<br>Participants were asked to complete the SCOFF questionnaire, which was scored by researchers. If the result indicated possible disturbed eating, the health professional was asked to complete a questionnaire and interview on their reaction and planned response, and their rating of the acceptability of screening.	Original	Not appropriate	C	Whilst the procedure and methodology of the study are appropriate and demonstrate a good use of the SCOFF as a screening tool, the SCOFF is not validated to detect DE.

Hautala et al. (2008)[118]	Prospective	To investigate the stability of self-reported ED symptoms, factors associated with them and the predictors of their recurrence.	SCOFF used to measure ED symptoms Participants were allocated to one of two groups depending on their SCOFF score - 'no symptoms' (0 points) and 'one or more symptoms' (1–5 points).	Finnish	Not appropriate	D	The SCOFF is a measure of EDs as a whole, not specific symptoms.
Herpertz-Dahlmann et al. (2008)[119]	Cross-sectional	To assess eating behaviour and to examine the association between body weight status and DE.	The SCOFF was used as a measure for DE amongst adolescents. Indicates that two positive answers from the SCOFF indicate DE behaviour and attitudes.	Original	Not appropriate	C	The SCOFF is not a valid measure of 'disordered eating', but of EDs.
Kaluski et al. (2008)[120]	Cross-sectional	To determine the prevalence of DE behaviours in a representative sample of Israeli adolescent school girls and to explain the different factors that prompt school girls to resort to DE behaviours.	SCOFF used to assess DE behaviours. Item 3 amended (1 stone (=6.35kg) reduced to 3 kg, to allow for the generally lower body weights of adolescents as compared to adults). The original questionnaire for the study was constructed in Hebrew and then translated into Arabic, for use within the Arab, Bedouin and Druze sectors. Back translation was carried out to ensure correct understanding and meaning.	Hebrew/Arabic	Not appropriate	C H	The SCOFF is not a valid measure of 'disordered eating', but of EDs.<br>Appropriate translation method but no validation of translated measure
Mond et al. (2008)[121]	Validation	To examine the comparative validity of the Eating Disorder Examination Questionnaire (EDE-Q) and SCOFF in screening for cases of EDs in a primary care sample of young adult women.	All participants who completed the SCOFF were approached to participate in a telephone interview, which included diagnostic items of the EDE, to assess for ED diagnosis. Minor changes were made to the wording of some questions used in the original (UK) version in order to accommodate use of the SCOFF in the US population.<br>Compared the validity of the EDE-Q and SCOFF in screening for cases of the more commonly occurring EDs in a primary-care setting.	Modified version of original	Appropriate		Appropriate validation method and description of the SCOFF as a screening measure.

Hautala et al. (2008)[122]	Cross-sectional	To examine gender differences in the prevalence of ED symptoms, in occurrence of health-risk factors and susceptibility to these and to investigate associations between health-risk factors and ED symptoms.	SCOFF used to measure ED symptoms. The outcome variable was dichotomised as 'no self-reported symptoms' (0 points) and 'one or more self-reported symptoms' (1–5 points).	Finnish	Not appropriate	D	The SCOFF is a measure of EDs as a whole, not specific symptoms.
Leung et al. (2009)[123]	Translation and Validation	To examine the validity and reliability of the SCOFF questionnaire for screening EDs in Hong Kong high school students.	The SCOFF was translated into Chinese using a back-translation method. Content and concurrent validity, test-retest reliability and internal consistency were all assessed. A score of 2 or more was interpreted as "indicating the potential of having an eating disorder".	Chinese	Appropriate		Appropriate translation and validation method.
Hautala et al. (2009)[124]	Cross-sectional	To evaluate the feasibility of the Finnish version of the SCOFF questionnaire in screening for ED symptoms among adolescents.	SCOFF used to measure ED symptoms. The authors gave clinical rationale behind a cut-off point of 1 for adolescent population. Allocated participants to one of two groups based on SCOFF score - 'no symptoms' (0 points) and 'one or more symptoms' (1–5 points).	Finnish	Not appropriate	D	The SCOFF is a measure of EDs as a whole, not specific symptoms.
Lähteenmäki et al. (2009)[125]	Validation	To test the validity of the SCOFF in a general population sample.	The SCOFF was used to screen current EDs, with a cut-off point of two or more positive answers. All participants who screened positive and a random sample of those who screened negatives were invited to a semistructured psychiatric interview (SCID), as a gold standard comparison.	Finnish	Appropriate		Appropriate validation method and use of the SCOFF as a screening tool.

Karukivi et al. (2010)[126]	Cross-sectional	To evaluate the association between alexithymia and ED symptoms in a nonclinical sample of late adolescents.	ED symptoms were assessed using the SCOFF questionnaire. A cut-off score of two or more points was used for classifying a subject as SCOFF positive.	Finnish	Not appropriate	D	The SCOFF is a measure of EDs as a whole, not specific symptoms.
Garcia et al. (2010)[127]	Translation & Validation	To translate and validate a French version of the SCOFF for the screening of ED in a student population	Back-translation method was used. After completing the SCOFF-F, each student was evaluated by one ED specialist blinded to SCOFF-F results. The validated French version of Mini International Neuropsychiatric Interview (MINI) and DSM-IV criteria for ED were employed as diagnostic references.	French	Appropriate		Appropriate translation and validation method
Toromanyan et al. (2007)[128]	RCT	To evaluate the effect of orally self-administered Slim339 (a dietary supplement)	SCOFF used to screen for 'abnormal eating behaviour'. Subjects with total scores of ≥2 were considered to be possible sufferers of AN or BN and were excluded from the trial.	Original	Mixed		Although the SCOFF generally should not be used to exclude participants from a control group (as it can include a high number of false positives), the authors of this study state that by scoring positive on the SCOFF a participant has a 'possibility' of having an ED. A stronger level of caution should ideally be used.
Wright et al. (2009)[129]	Cross-sectional	To examine the cooccurrence of DE and self-harm, onset timing, and the help-seeking of UK university students.	In methods section states SCOFF used to assess DE. Tended to report positive SCOFF screens as "possible ED cases".	Original	Mixed	E	The interpretation of the SCOFF is appropriate ('possible ED case'), however there is a lack of consistency as to whether the SCOFF is used to screen for EDs or DE.

Goldstone et al. (2009)[130]	Randomised cross-over	To investigate the interaction between nutritional state and the rewarding properties of food.	SCOFF used to exclude inappropriate individuals from the study. States all subjects had "normal eating habits" assessed via the SCOFF.	Original	Mixed	B	The SCOFF generally should not be used to exclude participants from a control group (as it can include a high number of false positives), however due to the SCOFF's high sensitivity, it could be reasonably assumed that anybody not screening positive on the SCOFF has 'normal eating habits'.
Hefner et al. (2009)[131]	Cross-sectional	To evaluate the relationship between mental health and social support in a large, random sample of college students.	The SCOFF was used to measure 'potential EDs'. Respondents who agreed with two or more statements were classified as having a possible ED and were reported as a 'positive ED screen'. In the discussion however, the authors repeatedly refer to 'symptoms of ED'	Original	Mixed	E<br>D	The SCOFF is described and used appropriately in the methodology of the study. However, the interpretation within the discussion is inappropriate. The SCOFF is a measure of EDs as a whole, not specific symptoms.
Ho et al. (2011)[132]	Cross-sectional	To investigate and compare the level of nutrition knowledge of health professionals, patients with ED and individuals without an ED as controls	The SCOFF was included to screen for latent EDs in participants. Classified participants into No ED (score of 0), Body Image/Food Issues (score of 1) and May Have ED (score of 2+)	Original	Not appropriate	F	There is no clinical rationale or validation of the scoring criteria (e.g., scoring interpretations of 0 & 1)

Rai et al. (2012)[133]	Cross-sectional	To estimate the burden of psychiatric and neurodevelopmental comorbidities in people with epilepsy.	The SCOFF questionnaire was used to screen for EDs. Defined people with a possible ED if they scored 2 or more (and answered affirmative to an additional item— "did your feelings about food interfere with your ability to work, meet personal responsibilities and/or enjoy a social life?"—capturing the potential impact on social functioning related to eating problems). Reported as 'screen-positive ED'.	Original	Appropriate		Appropriate level of caution and use of SCOFF as a screening tool.
Dooley-Hash et al. (2013)[134]	Cross-sectional	To describes patterns of emergency department utilisation by patients who screen positive for EDs.	Used to screen for EDs. Participants in the current study were given a modified version of the SCOFF - to increase understanding with US population and to capture BED. Reports results as 'screened positive/negative for an ED'	Modified version of original	Appropriate		Clear distinction of the results as a screen, however more caution needed ideally - e.g., 'screened positive for a possible ED'.
Leung et al. (2013)[135]	Open Trial	To identify the motivational stages of change in people with EDs using an Internet based self-help program developed in the Asia–Pacific region.	Participants identified themselves as suffering from EDs, and "met the criteria for an ED based on the SCOFF and the Eating Disorder Examination Questionnaire". An individual acquiring a score of 2 or above indicated a "high risk of ED"	Original	Not appropriate	B	Insufficient caution used when interpreting the results. Screening nature of the SCOFF not made clear.
Jantzer et al. (2013)[136]	Longitudinal	To explore whether adolescent children with a parent suffering from cancer show more externalizing and juvenile risk behaviours and whether reported behaviours develop differently over the course of 12 months in both groups.	The SCOFF was used to screen for eating disorders. Used a cut-off point of 2 or more for a positive screen and reported the results as 'occurrence of (ED) risk behaviours'.	Original	Not appropriate	B A	Not much detail given regarding the SCOFF scores however insufficient caution used when allocating participants to the 'occurrence of ED risk behaviour' group. The SCOFF has not been validated to assess a person's *risk* of developing an ED.

Eisenberg et al. (2012)[137]	Cross-sectional	To estimate mental health contagion, by applying an econometric identification strategy.	Uses SCOFF to assess ED risk. No description of the measure in the methods section and very limited reporting in the results.	Unknown	Not appropriate	A<br>G	The SCOFF has not been validated to assess a person's *risk* of developing an ED. As there is no description of the tool in the paper, it is unclear to the readers that the SCOFF is a screening measure.
Hochkogler et al. (2014)[138]	Cross-over	To explore the satiating effect of capsaicin analog, nonivamide, on subjective feelings of hunger, ad libitum food intake, and satiating hormones in moderately overweight male subjects	SCOFF was used to exclude the presence of EDs in participants.	Original	Not appropriate	B	Due to its screening nature, the SCOFF is not able to fully exclude EDs and such should not be used as an inclusion/exclusion criteria.
Watson et al. (2014)[139]	Cross-sectional	To describe the prevalence of screeningdetected EDs and DE in China and to explore the associations between dietary practices and DE.	DE was assessed by the SCOFF. A cut-off score of 2 or more was used to identify 'screening-detected ED'. The prevalence of DE was established by analysing responses on the five individual SCOFF items.	Chinese	Not appropriate	C	The use of the SCOFF to explore 'screening-detected eating disorders' is appropriate, although a clearer level of caution when interpreting the results would be ideal. However, using the individual items to assess DE is not appropriate. The SCOFF is not a valid measure of 'disordered eating', but of EDs.
Martinez-Gomez et a. (2015)[140]	Cross-sectional	To analyse the association between television viewing time and the risk of EDs, as well as the potential role of obesity in this association.	The risk of EDs was evaluated using the SCOFF. Answering positively ≥2 items of the SCOFF indicated a participant was at risk of ED.	Spanish	Not appropriate	A	The SCOFF has not been validated to assess a person's *risk* of developing an ED.

Peat et al. (2014)[141]	Cross-sectional	To explore the relationship between DE and media use in Chinese participants.	Authors analysed separate items on the SCOFF (vs. its composite score) in order to identify which specific ED symptoms (if any) were associated with media use.	Chinese	Not appropriate	D C	The SCOFF is a measure of EDs as a whole, not specific symptoms. The SCOFF is not a valid measure of 'disordered eating', but of EDs.
Hochkogler et al. (2016)[142]	RCT	To investigate whether a daily consumption of nonivamide in a proteinbased product formulation promotes a reduction in body weight in healthy overweight subjects and affects outcome measures associated with mechanisms regulating food intake	SCOFF used to exclude participants with EDs	Original	Not appropriate	B	Due to its screening nature, the SCOFF is not able to fully exclude EDs and such should not be used as an inclusion/exclusion criteria.
Piacentino et al. (2021)[143]	Cross-sectional	To assess the presence of Anabolic androgenic steroids and other misused substances in athletes' biological samples and link toxicological to psychopathological findings.	SCOFF appears to be used to assess prevalence of EDs, however limited information provided.	Original	Not appropriate	G	Insufficient information given regarding the use of the SCOFF and no description of its screening nature.
Galvao et al. (2020)[144]	Cross-sectional	To evaluate factors associated with ED symptoms among Brazilian adolescents.	ED symptoms were collected through the SCOFF. The outcome variable was evaluated as one continuous latent dimension of ED symptoms, ranging from 0 to 5 (the higher the score, the greater the amount of ED symptoms).	Original	Not appropriate	D	The SCOFF is a measure of EDs as a whole, not specific symptoms.

Purcell et al. (2015)[145]	Longitudinal	To test the assumptions that underpin the clinical staging model - a model that provides a longitudinal perspective of mental illness development.	SCOFF used to measure ED symptoms. Authors state that a total score of 2 or more indicates 'significant anorexia nervosa or bulimia nervosa symptoms'.	Original	Not appropriate	D	The SCOFF is a measure of EDs as a whole, not specific symptoms.
Purcell et al. (2015)[146]	Longitudinal	To present the baseline demographic and clinical characteristics of the Transitions Study cohort, particularly the nature and severity of psychopathology.	A score of 2 or more interpreted as 'probable caseness for an eating disorder'.	Original	Mixed		There is some level of caution used when interpreting the results, however ideally more emphasis would be made as to the screening nature of the tool.
Rothschild-Yakar et al. (2022)[147]	Cross-sectional	To validate and examine the potential of the Hebrew version of the Cambridge Mindreading face task to assess emotional theory of mind in individuals with AN.	Used to screen control subjects alongside the screening criteria of the structured clinical interview for DSM-IV Axis I DisordersPatient Edition. Controls were required to have no lifetime or current history of any psychiatric illness and no stigmata indicative of an ED.	Original	Not appropriate	B	Due to its screening nature, the SCOFF is not able to fully exclude EDs and such should not be used as an inclusion/exclusion criteria.
Castellon, Sudres & Voltzenlogel (2020)[148]	Cross-sectional	To investigate characteristics of selfdefining memories in females with AN.	SCOFF used to screen healthy controls. Participants were excluded from the healthy control group if they obtained 2 or more on the SCOFF.	French	Appropriate		There is limited information as to the interpretation of a participant scoring above the threshold on the SCOFF, however as they do not explicitly state they are excluding participants due to having an ED we can assume the use of the SCOFF is appropriate.

Gilon Mann et al. (2018)[149]	Cross-sectional	To compare threatrelated attention patterns of patients with AN restricting type, AN binge/purge type and healthy controls (n = 19).	Control participants were screened for EDrelated symptoms using the SCOFF. Excluded control participants answering positively on any one item of the SCOFF.	Original	Not appropriate	D	The SCOFF is a measure of EDs as a whole, not specific symptoms.
Zeeni et al. (2018)[150]	Cross-sectional	To investigate the use of different types of technology and media, attitudes toward them, and how they relate to physical and mental well-being in Lebanese university students.	SCOFF used to assess ED risk.	Original	Not appropriate	A	The SCOFF has not been validated to assess a person's *risk* of developing an ED.
Hei Chan, ZarateLopez & Martin (2021)[151]	Longitudinal	To evaluate symptom related outcomes of the FODMAP diet following group education and assess its effect on psychological profiles	SCOFF used to assess ED risk. A score of 2 or above indicated an increased risk of an ED.	Original	Not appropriate	A	The SCOFF has not been validated to assess a person's *risk* of developing an ED.
Maimon et al. (2018)[152]	Case-control	To compare the profiles of the two types of AN (restrictive and binge eating/purging) in terms of body composition, gynaecological status, disease history and the potential effects on bone metabolism.	SCOFF used to screen for history of ED in the control group.	Unknown	Not appropriate	A	Due to its screening nature, the SCOFF is not able to fully exclude EDs and such should not be used as an inclusion/exclusion criteria. It is also designed to measure current experiences and cannot assess historical presentations.

Saßmann et al. (2015)[153]	Cross-sectional	To examine the psychometric properties of the German version of the abbreviated 16-item Diabetes Eating Problem Survey–Revised (DEPSR) in a sample of young people with Type 1 diabetes.	Used as a comparison measure (along with EDE) to measure validity of the DEPS-R. The DEPS-R is a screening instrument designed to assess EDs in people with T1 diabetes. Describes SCOFF as a screening instrument designed to test for DE behaviour.	Original	Appropriate		As both the SCOFF and the DEPS-R appear to be screening tools for EDs then the use of the SCOFF in the validation process is appropriate. However, at times describes the SCOFF as measuring 'disordered eating'.
Alcaraz-Ibáñez et al. (2020)[154]	Cross-sectional	To examine the moderating/mediating role of social physique anxiety on the relationship between physical appearance comparisons and symptoms of DE in an adolescent population.	Symptoms of DE were assessed using the SCOFF. Threshold score of 2 or more used.	Spanish	Not appropriate	C	The SCOFF is not a valid measure of 'disordered eating', but of EDs.
Kenny & Carter (2018)[16]	Cross-sectional	To compare two cut-offs that have previously been used to differentiate clinical and subthreshold overvaluation of weight and shape using the EDE-Q.	Individuals were eligible for the NED (no history of ED) group if they reported never having been diagnosed with an ED and scored below a cut-off of 3 on the SCOFF.	Original	Not appropriate	F<br>B	No clinical rationale given for altered cut-off score. Due to its screening nature, the SCOFF is not able to fully exclude EDs and such should not be used as an inclusion/exclusion criteria. It is also designed to measure current experiences and cannot assess historical presentations.

Alcaraz-Ibáñez, Sicilia & Paterna (2019)[155]	Cross-sectional	To examine whether appearance/fitness social anxiety may independently contribute to explaining increased risk of ED and depressive symptoms in young adults	SCOFF used to assess risk of ED. Two or more positive responses indicated a high risk of ED.	Spanish	Not appropriate	A	The SCOFF has not been validated to assess a person's *risk* of developing an ED.
Evans et al. (2020)[156]	Qualitative	To explore the barriers for screening and integrated care of comorbid substance use and ED clients in a rural community substance use treatment setting.	During initial assessment, all clients would be screened using the SCOFF. Results of this and a medical screen would assist clinicians to determine acuity and a pathway of care for medical review or further assessment to determine the presence of ED or subthreshold eating concerns. Threshold score of 2 or more used.	Original	Appropriate		Appropriate use of the SCOFF as a screening measure. Further assessment completed before confirming presence of ED.
Simioni & Cottencin (2016)[157]	Feasibility	To estimate the feasibility and pertinence of implementing systematic screening for EDs in outpatient smoking cessation.	SCOFF included in the initial assessment in order to screening for EDs. 'Screen-positive patients' (i.e., SCOFF-F score ≥2) assessed for EDs by the physician using the MINI ED modules.	French	Appropriate		Appropriate use of the SCOFF as a screening measure. Further assessment completed before confirming presence of ED.
Aoun, Joundi & Gerges (2018)[158]	Cross-sectional	To examine the prevalence and correlates of a positive screen for EDs in a sample of Syrian refugees living in North Lebanon.	SCOFF was used to screen for EDs and assess risk of EDs. If two or more questions were answered positively, the screening for ED was considered positive and the individual was considered to be at 'high risk of developing ED'.	Arabic	Not appropriate	A	The SCOFF has not been validated to assess a person's *risk* of developing an ED.
Chan & Leung (2014)[159]	Validation	To validate an online version of the Eating	Examined convergent validity of the online EDE-Q with the SCOFF Questionnaire (and	Original	Appropriate		There is an inaccurate interpretation of the SCOFF as

		Disorder Examination Questionnaire (EDE-Q) in a sample of university students in Hong Kong.	the Body Shape Questionnaire). A score ≥2 in the SCOFF was indicative of a high risk of having an ED.				measuring a person's 'risk of having an ED'. In terms of the SCOFFs use within the methodology of this study, as the online EDE-Q is being proposed as a screening measure for EDs, the SCOFF would be an appropriate tool for convergent validity.
Giel et al. (2016)[160]	Cross-sectional	To investigate eating disorder pathology in German elite adolescent athletes.	Athletes were classified as showing ED pathology when two or more SCOFF questions were answered affirmatively. In the results and discussion section reported results as 'positive SCOFF screening', 'ED symptoms' and 'ED pathology'.	Original	Not appropriate	E	Insufficient and inconsistent caution used when reporting the SCOFF results.
Kenny, Singleton & Carter (2017)[161]	Cross-sectional	To explore the relationship between emotion regulation and binge eating disorder.	Used as an exclusion criterion for the study. Participants who exceeded a cut-off of 3 were not eligible to be included in the NED (no history of ED) group.	Original	Not appropriate	B	Due to its screening nature, the SCOFF is not able to fully exclude EDs and such should not be used as an inclusion/exclusion criteria. It is also designed to measure current experiences and cannot assess historical presentations.
Patmore, Meddaoui & Feldman (2019)[162]	Case-study	To illustrate the utility, relevance, and effectiveness of cognitive behavioural therapy on alleviating ED symptoms in an adult Latina patient.	The participant was assessed and diagnosed with an ED using both formal and informal means; this included a clinical interview, the Patient Health Questionnaire-9 and the SCOFF questionnaire. Given the frequency of binge and purge episodes and a score of 3 on the SCOFF, the participant was diagnosed with BN.	Original	Not appropriate	B G	Insufficient caution used when interpreting the SCOFF scores. Although a clinical interview was also used, there is no clear description of the screening nature of the SCOFF and could be interpreted as being used diagnostically.

Peters, Bowen & Balbuena (2019)[163]	Cross-sectional	To determine whether mood instability in people with anxiety disorders contributes to trait impulsivity, nonsuicidal self-injury, and binge eating/purging.	The presence of binge eating/purging (BE/P) in the past year was assessed with the SCOFF screening questionnaire. Authors defined the presence of BE/P as a 'Yes' response to items 1 and 2.	Original	Not appropriate	D	The SCOFF is a validated measure to screen for AN and BN. It should be used as a whole measure and not in part. It has not been validated to measure binge-eating or purging behaviours individually.

Robert et al. (2020)[164]	Cross-sectional	To assess whether optimism is associated with body mass index (BMI), eating behaviour and EDs in a populationbased study	SCOFF was used to identify EDs. An overall score ≥ 2 indicates ED risk.<br>The Expali algorithm was used to distinguish the different ED categories, taking into account each SCOFF response and the individual's BMI to classify participants in four broad categories.	French	Not appropriate	B A	The SCOFF questionnaire is designed as a screening measure. The categories of ED used in the Expali algorithm were derived from DSM-5, however the SCOFF validated in line with the DSM-IV criteria.<br>The SCOFF has not been validated to assess a person's *risk* of developing an ED.
Strand et al. (2019)[165]	Cross-sectional	To explore the prevalence of symptoms of DE and body image concerns among international adoptees.	Symptoms of DE were measured by the Three-Factor Eating Questionnaire (TFEQr21) and parts of the SCOFF scale. Included items 1, 2 and 5 of the SCOFF.	Original	Not appropriate	C	The SCOFF is not a valid measure of 'disordered eating', but of EDs. It should be used as a whole measure and not in part.
Van Dyke & Drinkwater (2022)[166]	Cross-sectional	To investigate the prevalence of intuitive eating, and associations between intuitive eating and indicators of physical and mental health, among a general population of rural adults.	SCOFF was used to measure and control for indication of an ED.	Original	Appropriate		Limited detail regarding interpretation of SCOFF scores provided. Some level of caution used with the term 'indication', however ideally further caution and clarification would be included.

Khalil et al. (2022)[167]	Cross-sectional	To evaluate the psychological impact of the lockdown in Lebanon	SCOFF used to measure EDs. Cut-off score of two or more used. Does not interpret/give meaning to SCOFF scores, simply reports them as "SCOFF score".	Original	Mixed	B	Very limited information or interpretation of SCOFF tool and scores.
Baechle et al. (2019)[168]	Longitudinal	To analyse the prevalence and course of DEB in adolescents with intensively treated type I diabetes.	A diabetes-adapted version of the SCOFF questionnaire was used to assess ED symptoms. In the adapted version, item 5 was replaced with a question on insulin restriction ("How often did you inject too little or no insulin after carbohydrate intake during the last week?"; cut-off at least almost every day). Authors stated that because screeningpositive results were not validated with diagnostic interviews, the term "DEB" was used instead of EDs.	Modified version of original	Not appropriate	C	Clinical rationale given as to alteration of item wording. Whilst acknowledgement is made to the SCOFF's inability to diagnose EDs, the SCOFF is not a measure of 'disordered eating'. A level of caution when interpreting results as EDs would be preferred.
Bryant et al. (2021)[169]	Validation / Pilot Study	To describe the development and pilot validation of a novel digital screening tool, the InsideOut Institute-Screener (IOIS), to assess eating disorder risk and sub threshold illness.	SCOFF used to assess the concurrent validity and convergent validity of the IOI-S. Also used in the development and writing of the tool.	Original	Appropriate		The IOI-S measures a similar concept to the SCOFF, however the SCOFF has not been validated to assess a person's *risk* of developing an ED. Nevertheless, as the SCOFF is used among other validated tools, it can be deemed appropriate.
Ganson & Nagata (2021)[170]	Cross-sectional	To determine the associations between vaping and a selfreported lifetime ED diagnosis and ED risk among a large sample of college students.	SCOFF used to measure ED risk. Elevated ED risk was indicated by a score of ≥2 "yes" responses	Original	Not appropriate	A	The SCOFF has not been validated to assess a person's *risk* of developing an ED.

Ganson et al. (2022)[171]	Cross-sectional	To determine the association between lifetime use of legal and illegal appearance- and performance-enhancing drugs and ED symptomatology among a large, non-clinical sample of college students.	ED symptomatology was measured using the SCOFF. A positive ED screen was determined by a score of two or more "yes" responses, resulting in a dichotomous variable (negative versus positive screen)	Original	Not appropriate	B E	Whilst the results of the SCOFF are reported correctly (i.e., positive ED screen), the interpretation of what these results measure is somewhat lacking in caution (i.e. ED symptomatology)
Lenk et al. (2021)[172]	Cross-sectional	To examine a wide range of psychopathological and socioeconomic factors to identify those primarily associated with periodontitis.	Used to assess ED symptoms. No details given as to cut-off score used.	Original	Not appropriate	D<br>G	The SCOFF is a measure of EDs as a whole, not specific symptoms.<br>Insufficient detail given regarding use of the measure.
Lichtenstein et al. (2021)[173]	Validation	To examine the factor structure and psychometric properties of the EDE-Q in Danish.	Used to assess construct validity of the Danish version of EDE-Q.	Danish	Appropriate		Appropriate use of the SCOFF as a screening tool, as the EDE-Q is a screening tool measuring similar concepts.
Lommi et al. (2020)[174]	Validation	To validate the Children's Eating Attitudes Test (ChEAT) in the Finnish population	SCOFF was used to assess the concurrent validity. However, both the SCOFF and the ChEAT are described as measuring DE symptoms. Uses a cut-off score of 2 to indicate DE symptoms.	Original	Not appropriate	C	As the SCOFF does not measure disordered eating symptoms, but EDs as a whole, it is not an appropriate tool to assess concurrent validity of this measure.

Maguen et al. (2018)[175]	Cross-sectional	To develop a primary care ED screen, Screen for Disordered Eating (SDE), with greater accuracy and greater potential for generalizability, compared to existing screens.	SCOFF used to evaluate the discriminative accuracy of the new screen. Also compared sensitivity, specificity, PPV and NPV.	Original	Appropriate		Appropriate use of the SCOFF as a screening tool, as the SDE is a screening tool measuring similar concepts. Whilst the name of the measure suggests it assesses DE, from the description it appears to be screening for EDs.
Robert et al. (2022)[176]	Longitudinal	To assess cross-sectional and longitudinal associations between resilience and ED in a large cohort of French adults.	ED symptoms were measured with the SCOFF. A total score ≥2 indicated ED symptoms.	French	Not appropriate	D	The SCOFF is a measure of EDs as a whole, not specific symptoms.
Vila-Martí, Elío & Sumalla-Cano (2021)[177]	Protocol/Prospective	To describe the protocol of a study to determine the eating behaviour and predisposition to suffer EDs in first-year college students.	Used to assess the risk of presenting with an ED.	Original	Not appropriate	A	The SCOFF has not been validated to assess a person's *risk* of developing an ED.
Zeiler et al. (2021)[178]	Network analysis	To investigate psychopathological symptoms and wellbeing/quality of life in overweight or obese adolescents from a large representative community sample.	Used to assess ED risk. A score ≥ 2 represents an increased risk for eating disorders.	Original	Not appropriate	A	The SCOFF has not been validated to assess a person's *risk* of developing an ED.

Tavolacci, Ladner & Déchelotte (2021a) [179]	Longitudinal	To identify the changes in body mass index and EDs among university students between 2009 and 2021.	Used the Expali algorithm to assess prevalence of EDs.	French	Not appropriate	B	The SCOFF questionnaire is designed as a screening measure. The categories of ED used in the Expali algorithm were derived from DSM-5, however the SCOFF validated in line with the DSM-IV criteria.
Martin et al. (2020)[180]	Cross-sectional	To examine the relationship between inattentive and hyperactive/impulsive symptoms of ADHD and DE behaviour.	Used the SCOFF to assess DE/risk of ED. Participants assigned to 'low risk' or 'high risk' groups depending on SCOFF score.	Original	Not appropriate	A	The SCOFF has not been validated to assess a person's *risk* of developing an ED.
Šablatúrová et al. (2021)[181]	Cross-sectional	To study the association between excessive internet use and ED through their common underlying psychological factors: internalising problems and externalising problems	Used to assess ED symptoms and ED risk along with 2 items from the ED screen for primary care targeted at BN.	Original	Not appropriate	A	The SCOFF has not been validated to assess a person's *risk* of developing an ED.
Tavolacci, Ladner & Déchelotte (2021b)[182]	Cross-sectional	To identify factors, such as changes in food choices, lifestyle, risk and protective behaviour, mental health, and social demographics, on EDs.	Used the Expali algorithm to assign participants into 4 broad categories of EDs.	French	Not appropriate	B	The SCOFF questionnaire is designed as a screening measure. The categories of ED used in the Expali algorithm were derived from DSM-5, however the SCOFF validated in line with the DSM-IV criteria.

Romo et al. (2018)[183]	Cross-sectional	To determine the possible links between attention-deficit hyperactivity disorder and the presence of concomitant addictions with or without substance.	Used to assess eating behaviours, however in the results section, interprets a positive SCOFF screen as participants 'suffer[ing] from an ED'.	Original	Not appropriate	B	Insufficient caution used when interpreting the SCOFF results.
Trott et al. (2021)[184]	Validation	To validate a novel screening tool able to stratify between primary and secondary exercise addiction, called the secondary exercise addiction scale (SEAS).	Used to assess predictive value, concurrent validity, sensitivity and specificity of the eating disorder section of the SEAS.	Original	Appropriate		Appropriate use of the SCOFF as a screening tool, as the SEAS is a screening tool measuring similar concepts.
Zickgraf, Hazard & O'Connor (2022)[185]	Cross-sectional	To examine the association between food insecurity and ED risk independent of cooccurring anxiety/depression.	In the methods section, the SCOFF is described as being used to assess ED symptoms, however in the results section reports a positive SCOFF screen as participants 'meeting criteria for probable ED'.	Original	Appropriate		A more consistent description of the SCOFF questionnaire would be ideal, however the results are interpreted correctly.
Sidani et al. (2016) [186]	Cross-sectional	To examine the association between social media use and eating concerns in a large, nationally representative sample of young adults.	Participants were presented with five items that were adapted from the SCOFF and the ED Screen for Primary Care (ESP). Items were formatted as statements with a 5-point Likert-type scale. Scores were put into 'low', 'medium', 'high' eating concerns.	Original (adapted)	Not appropriate	F	No clinical rationale or validation for amended items and scoring.

Kotyuk et al. (2018)[187]	Protocol	To describe the protocol of the Psychological and Genetic Factors of Addictive Behaviours (PGA) study, which applies an integrative approach to understanding the acquisition, development, and maintenance of addictive behaviours.	Used to screen for/detect EDs. Limited information regarding how the results will be interpreted.	Original	Appropriate		Appears appropriate from the protocol, however further information would be required regarding the interpretation of the results and exactly what the SCOFF is being used to measure.
Gruneis et al. (2021)[188]	Randomised cross-over	To investigate the effect of the sweetness of a sucrose versus an isocaloric glucose solution in dietary	SCOFF used to exclude those with EDs.	Original	Not appropriate	B	Due to its screening nature, the SCOFF is not able to fully exclude EDs and such should not be used as an inclusion/exclusion criteria.
		concentrations on blood glucose regulation.					
Tseng et al. (2016)[189]	Cross-sectional	To investigate the influence of clinical characteristics on presenting symptoms of patients with EDs.	All participants were asked to complete the SCOFF and were interviewed blindly by one of two research assistants using the ED module of the Structured Clinical Interview for DSM-IV-TR Axis I Disorders, Patient Version (SCID-I/P).	Mandarin	Appropriate		Appropriate use of the SCOFF as a screening measure before conducting further assessment.
Monterrosa Castro, Beltrán-Barrios & Mercado-Lara (2021)[190]	Cross-sectional	To identify the frequency of sleep complaints and associated menopausal symptoms.	Used to screen for probable ED. Two or more points indicated probable ED.	Original	Appropriate		Some level of caution when interpreting the results, but clearer identification of the results as a screen would be idea.

Herpertz-Dahlmann et al. (2014)[191]	Longitudinal	To investigate the stability of eating disordered behaviour, its effect on the development of over- and underweight, and its contribution to adverse outcomes in mental health, especially depressive states in young adulthood	Eating-disordered behaviour was assessed by the SCOFF questionnaire. Some modifications were made to the language to account for cultural differences. A positive screening status was defined as 2 or more positive answers. Results interpreted as 'prevalence of disordered eating', 'disturbed eating' and 'eating disordered behaviour'.	Original	Not appropriate	B	The SCOFF is not a valid measure of 'disordered eating', but of EDs and should not be used to measure prevalence.
